# A Polar Sulfamide Spacer Significantly Enhances the Manufacturability, Stability, and Therapeutic Index of Antibody–Drug Conjugates

**DOI:** 10.3390/antib7010012

**Published:** 2018-02-20

**Authors:** Jorge M. M. Verkade, Marloes A. Wijdeven, Remon van Geel, Brian M. G. Janssen, Sander S. van Berkel, Floris L. van Delft

**Affiliations:** Synaffix BV, Industrielaan 63, 5349 AE Oss, The Netherlands; j.verkade@synaffix.com (J.M.M.V.); m.wijdeven@synaffix.com (M.A.W.); r.vangeel@synaffix.com (R.v.G.); b.janssen@synaffix.com (B.M.G.J.); s.vanberkel@synaffix.com (S.S.v.B.)

**Keywords:** antibody–drug conjugates (ADCs), therapeutic index, spacer technology, carbamoyl sulfamide, chemoenzymatic, glycan-remodeling, copper-free click chemistry

## Abstract

Despite tremendous efforts in the field of targeted cancer therapy with antibody–drug conjugates (ADCs), attrition rates have been high. Historically, the priority in ADC development has been the selection of target, antibody, and toxin, with little focus on the nature of the linker. We show here that a short and polar sulfamide spacer (HydraSpace™, Oss, The Netherlands) positively impacts ADC properties in various ways: (a) efficiency of conjugation; (b) stability; and (c) therapeutic index. Different ADC formats are explored in terms of drug-to-antibody ratios (DAR2, DAR4) and we describe the generation of a DAR4 ADC by site-specific attachment of a bivalent linker–payload construct to a single conjugation site in the antibody. A head-to-head comparison of HydraSpace™-containing DAR4 ADCs to marketed drugs, derived from the same antibody and toxic payload components, indicated a significant improvement in both the efficacy and safety of several vivo models, corroborated by in-depth pharmacokinetic analysis. Taken together, HydraSpace™ technology based on a polar sulfamide spacer provides significant improvement in manufacturability, stability, and ADC design, and is a powerful platform to enable next-generation ADCs with enhanced therapeutic index.

## 1. Introduction

It took more than 50 years since the pioneering studies by Mathé et al. on targeted tumor treatment with methotrexate for the first antibody–drug conjugate (ADC), Adcetris^®^, to reach market approval in 2011. Since then, three more ADCs were approved or re-approved, i.e., Kadcyla^®^ (in 2013), Besponsa^®^ (2017), and Mylotarg^®^ (2017). Mechanistically, ADCs exert their activity by (a) selective binding of antibody to tumor; (b) internalization; and (c) lysosomal degradation and liberation of toxic payload, leading to cytotoxic cell death [1]. In recent years, however, evidence has emerged that conjugation technology is also a critical component in determining quality attributes of an ADC [2,3]. For example, although the majority (~65%) of clinical ADCs are based on random payload attachment [4], a clear trend toward site-specifically conjugated ADCs based on engineered cysteine can be noted (currently >10 in the clinic), generally showing an increased therapeutic index [5,6]. A range of other site-specific conjugation technologies has been explored in the past decade [1,4,7,8,9], but to date only genetic encoding [10] of a non-natural amino acid (*p*-acetophenylalanine), suitable for oxime ligation, has reached clinical status (two ADCs).

Besides conjugation technology, the nature of the linker connecting the antibody and the toxic payload also determines ADC properties, particularly in light of the high hydrophobicity of (nearly) all cytotoxic payloads, which may negatively impact manufacturability, stability (aggregation) [11], pharmacokinetics [12], and therapeutic index [13]. To offset payload hydrophobicity, Immunogen introduced onto the linker of DM4 a negatively charged sulfonyl group [14,15]: for example, in phase III, the folate receptor-targeting ADC IMGN853 (mirvetuximab soravtansine). Introduction of an extended polyethylene glycol (PEG) spacer [16,17] is also partially effective, but may concomitantly ameliorate ADC pharmacokinetics [13,18], unless present as a side-chain [19]. Alternative approaches to increase linker polarity, such as a glucuronide [19] or a pyrophosphate [20], are still at the preclinical stage.

**Figure 1:**
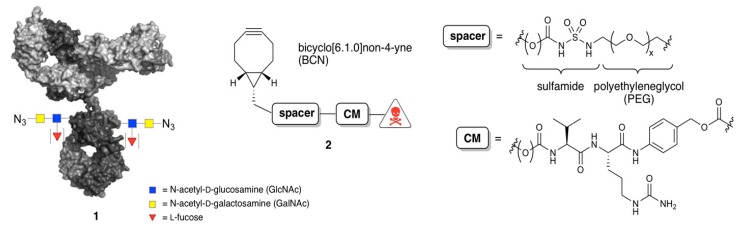
Generic structures of azidosugar-functionalized antibody (**1**) and BCN-spacer-CM-payloads (**2**). The spacer may consist solely of a polyethyleneglycol (PEG) chain or may be additionally modified, as described herein, with acyl/carbamoyl sulfamide (jointly called HydraSpace™). In addition, construct **2** may contain a protease-sensitive cleavable moiety (CM), typically consisting of Val-Cit-*p*-aminobenzyloxycarbonyl (vc-PABC), as depicted.

**Figure 2:**
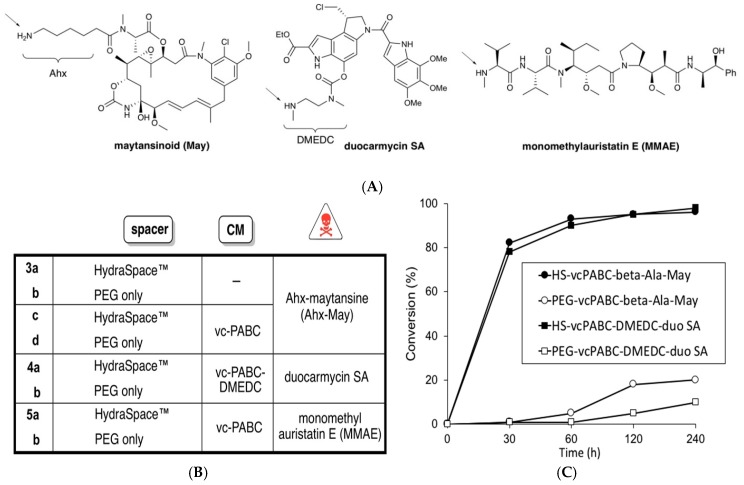
(**A**) Structures of payloads with point of attachment indicated with arrow. The 6-aminohexanoyl (Ahx) remains bound to the maytansinoid after release, while the *N*’,*N*-dimethylethylenediaminocarbonyl (DMEDC) moiety on duocarmycin SA is designed for self-elimination by cyclization; (**B**) BCN constructs used in this study for conjugation to trastuzumab-**1** or brentuximab-**1**; (**C**) Conjugation efficiency of trastuzumab-**1** with BCN-payload constructs based on maytansinoid (May) or duocarmycin SA (duo SA), with PEG only (PEG) or with sulfamide moiety (HS). In each case, three equivalents of BCN-payload were used with regard to trastuzumab-**1** (1.5 equivalent per azide).

All monoclonal antibodies are glycosylated at (or around) the same asparagine (Asn^300^) residing in the C_H_2 domain. As such, the globally conserved glycan provides a convenient natural anchor point for preparation of antibody conjugates [21,22]. We recently described [23] a versatile strategy for the preparation of homogeneous ADCs with tailored DAR (2 or 4) based on enzymatic trimming of the antibody glycan to the *N*-acetylglucosamine moiety attached directly to N^300^ prior to payload attachment (GlycoConnect™ technology, Figure 1). In short, GlycoConnect™ entails (a) introduction of an azidosugar onto the antibody core GlcNAc (**1**) by trimming and glycosyl transfer in a one-pot process and (b) metal-free click conjugation [24] of a toxic payload (**2**). While earlier studies relied partially on the conventional PEG spacers, we here show that GlycoConnect™ technology is further enhanced by introduction of a readily installed, polar acyl or carbamoyl sulfamide moiety (HydraSpace™). It is demonstrated that for a range of different payloads, with cleavable or non-cleavable linker (Figure 2A,B), introduction of a carbamoyl sulfamide moiety versus “PEG-only” spacers favorably impacts (a) manufacturability; (b) stability; and (c) in vivo performance of the resulting ADC. Head-to-head comparison of two HydraSpace™-containing ADCs with marketed products Adcetris^®^ and Kadcyla^®^ indicated a significant improvement in the efficacy and safety in several in vivo models, and was corroborated by in-depth pharmacokinetic analysis. Based on these findings, the HydraSpace™ technology constitutes a promising platform for next-generation ADCs with significantly enhanced manufacturability, stability, and, particularly, therapeutic index.

## 2. Materials and Methods

### 2.1. Synthesis of BCN-Spacer-Payload Constructs

Chemical preparation of HydraSpace™-containing compounds from bicyclo(6.1.0)non-4-yne (BCN) alcohol proceeded according to the general scheme depicted in Figure S1. Full synthetic procedures for all BCN-spacer-payload constructs **3a**–**d**, **4a**–**b**, **5a**–**b**, **6**, and **7** can be found in the Supplementary Materials.

### 2.2. Remodeling and Conjugation Procedure for Conversion of Antibodies into ADCs

Remodeling of trastuzumab for preparation of DAR2 ADCs incorporating **3a**–**d**, **4a**–**b** was performed by (a) trimming with endoglycosidase and (b) enzymatic transfer of GalNAz under the action of GalT(Y289L), followed by copper-free click conjugation of toxic payload, largely according to our earlier described methodology [23]. For preparation of both DAR4 ADCs, incorporating **6** or **7**, respectively, as well as for brentuximab DAR2 ADC incorporating **5a**–**b**, a procedure was applied involving the transfer of a 6-azidoGalNAc substrate under the action of β-(1,4)-*N*-acetylgalactosaminyl transferase (manuscript in preparation). Conversions were determined by RP-HPLC after DTT reduction (as previously described) [23] as exemplified in Figure S7 for conversion trastuzumab → trastuzumab-**1** → conjugation with **6**. Mass spectrometric data of ADCs are summarized in Table S1, obtained after DTT reduction or after IdeS digestion (MS analysis of Fc/2 fragment), as previously described [23]. For IdeS digestion, a solution of 20 µg (modified) IgG was incubated for 1 h at 37 °C with IdeS (1.25 U/µL), commercially available as Fabricator™ from Genovis, Lund, Sweden, in phosphate-buffered saline (PBS) pH 6.6 in a total volume of 10 µL. Samples were washed three times with MilliQ using an Amicon Ultra-0.5, Ultracel-10 Membrane (Millipore), resulting in a final sample volume of approximately 40 µL. Next, the samples were analyzed by electrospray ionization time-of-flight (ESI-TOF) on a JEOL AccuTOF. Deconvoluted spectra were obtained using Magtran software.

## 3. Results

### 3.1. Studies of Conjugation Rates

Trastuzumab-**1** was conjugated with BCN-linker-payload constructs according to the general procedure described in the materials and methods Section 2.2. Specifically, trastuzumab-**1** (15 mg/mL in phosphate-buffered saline (PBS) pH 7.4) was incubated with the appropriate BCN-linker-payload construct (1.5 equiv./azide) in the presence of a co-solvent (5–12.5% of *N*,*N*,-dimethylacetamide (DMA) or *N*,*N*-dimethylformamide (DMF)) containing the BCN-linker-payload (1.5 equiv./azide). Samples were taken at time points 0, 30, 60, 120, and 240 min. Conversions were estimated by mass spectral analysis and plotted for BCN-linker-payload constructs based on maytansinoid or duocarmycin, and containing a cleavable moiety (Figure 2C), as described in the Supplementary Materials.

### 3.2. Xenograft Studies with HER2-Overexpressing Models HBCx-13B or T226

**Figure 3:**
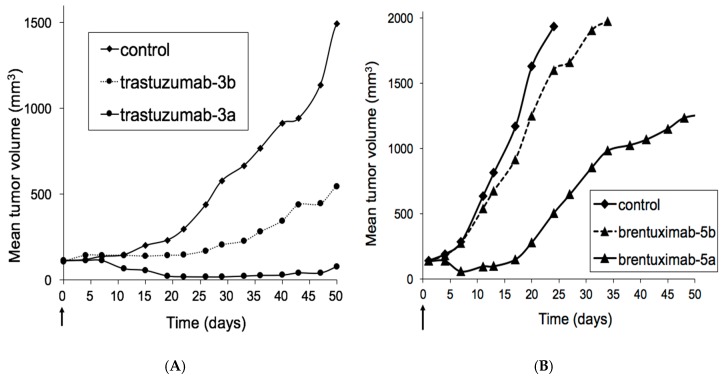
(**A**) In vivo efficacy of HydraSpace™-derived **3a** or PEG only variant **3b** in HBCx-13B patient-derived xenograft (PDX). A single dose of either ADC (6 mg/kg) was administered on *t* = 0 and tumor size was monitored over time (*n* = 5); (**B**) In vivo efficacy in Karpas-299 xenograft (CDX) of ADCs derived from brentuximab-**1** conjugated with either HydraSpace™ BCN-MMAE construct **5a** or PEG-only variant **5b**. A single dose of either ADC (1 mg/kg) was administered on *t* = 0 and tumor size was monitored over time (*n* = 8).

**Figure 4:**
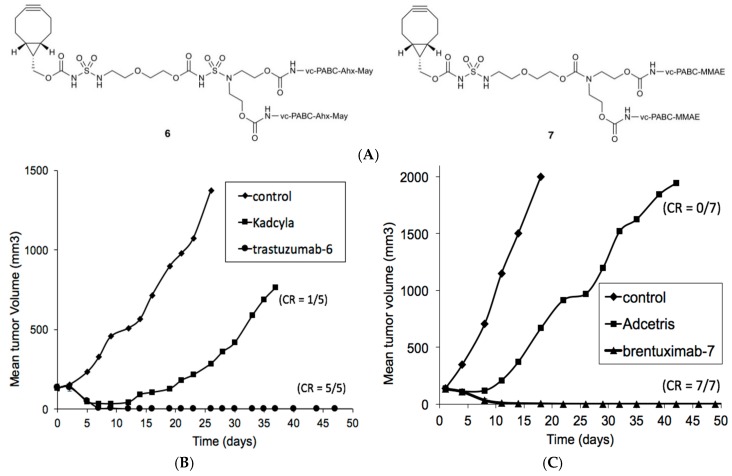
(**A**) Structures of branched HydraSpace™ BCN-payload constructs **6** and **7**; (**B**) In vivo efficacy in T226 patient-derived xenograft (PDX) of GlycoConnect™ ADC derived from trastuzumab-**1** conjugated with HydraSpace™ BCN-maytansine construct **6** versus Kadcyla^®^. A single dose of ADC (9 mg/kg) was administered on *t* = 0 and tumor size was monitored over time (*n* = 5). Number of complete responders (CR) is indicated in the graph. (C) In vivo efficacy in Karpas-299 cell-derived xenograft (CDX) of GlycoConnect™ ADC derived from brentuximab-**1** conjugated with HydraSpace™ BCN-MMAE construct **7** versus Adcetris^®^. A single dose of either ADC (1 mg/kg) was administered on *t* = 0 and tumor size was monitored over time (*n* = 7). Number of complete responders (CR) is indicated in the graph.

Female athymic nude mice and female SCID Hairless Outbred (SHO) mice (Hsd:Athymic Nude-Fox1nu and Crl:SHO-Prkdcscid Hrhr, six to nine weeks old at the beginning of the experimental phase, obtained from Charles River Laboratories, L’Arbresles, France) were anesthetized with ketamine/xylazine; the skin was aseptized with a chlorhexidine solution, incised at the level of the interscapular region, a 20-mm^3^ tumor fragment 20 (HBCx-13B or T226 breast cancer patient-derived xenograft model) was placed in the subcutaneous tissue, and the skin was closed with clips. When the tumor volume was in the range of 60 to 200 mm^3^, mice were injected intravenously (i.v.) with either vehicle (control), trastuzumab-**3a** (at 6 mg/kg), trastuzumab-**3b** (at 6 mg/kg), Kadcyla^®^ (at 9 mg/kg) or trastuzumab-**6** (at 9 mg/kg) and tumors were measured twice weekly as depicted in Figure 3A (HBCx-13B xenograft in athymic nude mice) and Figure 4B (T226 xenograft in SHO mice).

### 3.3. Xenograft Studies with Karpas-299 Model

Female CB.17 SCID mice (eight to 12 weeks old at the beginning of the experimental phase, obtained from Charles River Laboratories, Wilmington, MA, USA) were injected with 1 × 10^7^ Karpas-299 tumor cells in a 50% Matrigel subcutaneous in the flank (Karpas-299 cell xenograft model). When the tumor volume was in the range of 100–150 mm^3^, groups of eight mice were injected i.v. with a single dose of either vehicle (control), brentuximab-**5a** or brentuximab-**5b** (all at 1 mg/kg) and tumors were measured twice weekly as depicted in Figure 3B. In another study, groups of seven mice were injected i.v. with a single dose of either vehicle, Adcetris^®^ or brentuximab-**7** (all at 1 mg/kg) and tumors were measured twice weekly as depicted in Figure 4C.

### 3.4. Tolerability Studies of Trastuzumab-**6** and Kadcyla^®^

Sprague-Dawley rats (two females per group), six weeks old at the beginning of the experimental phase, obtained from Charles River Laboratories, Wilmington, MA, USA, were treated by intravenous (bolus) injection using a microflex infusion set introduced into a tail vein (2 mL/kg at 1 mL/min) with trastuzumab-**6** or Kadcyla^®^ (at 20, 35, 50, or 60 mg/kg). One group of animals was treated with the vehicle (control). After dosing, all animals were maintained for a 12-day observation period. Surviving animals were killed on day 12. Each animal was weighed at the time of randomization/selection, prior to dosing (day 0) and on all consecutive days of treatment.

### 3.5. Tolerability Studies of Brentuximab-**7** and Adcetris^®^

CR female Wistar rats (2 females per group), 5–6-week-old at the beginning of the experimental phase, obtained from Charles River Laboratories, Wilmington, MA, USA, were treated with brentuximab-**7** (at 40 mg/kg, 60 mg/kg, 70 mg/kg, and 80 mg/kg) and compared to Adcetris^®^ (at 15 mg/kg, 20 mg/kg, and 40 mg/kg). The test items were administered via intravenous (bolus) injection using a microflex infusion set introduced into a tail vein (2 mL/kg at 1 mL/min). One group of animals was treated with the vehicle (control). After dosing, all animals were maintained for a 12-day observation period. Surviving animals were euthanized on day 12. Each animal was weighed at the time of randomization/selection, prior to dosing (day 0) and on all subsequent days up to day 12. Any individual animal with a single observation of > than 30% body weight loss or three consecutive measurements of >25% body weight loss was euthanized.

### 3.6. Pharmacokinetics Studies of Anti-HER2 ADCs

For the determination of conjugated drug, a commercially available kit for the determination of DM1 antibody–drug conjugates was used (Eagle Biosciences, Nashua, NH, USA). The Eagle Biosciences DM1 Antibody–Drug Conjugate (ADC) ELISA Assay Kit is designed, developed, and produced for the quantitative measurement of antibody DM1 conjugate (trastuzumab emtansine) in serum, tissue, and cell culture samples. The assay utilizes the competitive immunoassay technique with an antibody that exclusively binds to maytansinoids.

Samples are added to wells of a microtiter plate that is coated with specific anti-DM1 antibody. Subsequently, a horseradish peroxidase (HRP)-conjugated DM1 is added to each well. During the incubation period, the antibody–DM1 conjugate competes with the HRP-conjugated DM1 for the limited binding sites of anti-DM1 antibody. An immune complex of well-coated “anti-DM1 antibody–HRP-conjugated DM1” is formed. The unbound antibodies and buffer matrix are removed in the subsequent washing step. For the detection of this immunocomplex, the well is then incubated with a substrate solution in a timed reaction, which is terminated with an acidic reagent (i.e., ELISA stop solution). The absorbance is then measured in a spectrophotometric microplate reader. The enzymatic activity of the immunocomplex bound to the wall of each microtiter well is inversely proportional to the amount of antibody–DM1 conjugate in the test sample. A calibration curve is generated by plotting the absorbance versus the respective antibody–DM1 conjugate concentration for each calibrator on a four-parameter or log-logit curve fitting. The concentration of antibody–DM1 conjugate in test samples is determined directly from this calibration curve.

## 4. Discussion

We set out to explore the sulfamide moiety (Figure 1) as a putative functionality for short and polar spacers. While both acyl and carbamoyl sulfamides are applied in medicinal chemistry as an anionic pharmacophore [25], in particular carbamoyl sulfamides can be constructed in a one-pot procedure from alcohols and amines (Figure S1). Thus, HydraSpace™-containing compounds **3a**,**c** and **4a** (Figure 2B) were readily obtained from bicyclo(6.1.0)nonyne (BCN) alcohol [24] via addition of chlorosulfonyl isocyanate (CSI), a short spacer and a maytansinoid or duocarmycin SA derivative. As maytansinoid payloads are known to be effective both with a non-cleavable linker (e.g., DM1 in Kadcyla^®^) and with a cleavable linker (e.g., DM4 in mirvetuximab soravtansin), both BCN-maytansinoid constructs were prepared as in **3a** (non-cleavable) and **3c** (cleavable). For duocarmycin SA, only the version with the requisite cleavable moiety (vc-PABC-DMEDC, as in **4a**) was synthesized, as well for all BCN payload constructs the analogous variants based on “PEG-only” (**3b**,**d** and **4b**). The first indication of difference in polarity between “HS” constructs versus “PEG-only” constructs became apparent by a significantly shorter HPLC retention time (~1.5 min, Figure S2). We attribute this shorter retention time to deprotonation of the central amine in the acylated sulfamide moiety R^1^C(O)NHSO_2_NHR^2^ (reported pKa 2–5, depending on R^1^ and R^2^) [25], thus generating a highly polar anionic compound at neutral pH. The crucial role of the central amino group in determining the polarity was further corroborated by retention time determination of a set of model sulfamides based on BCN and benzylamine only, in acidic and neutral eluent (Figure S3).

Next, we explored the conjugation efficiency of azidosugar-remodeled antibody (**1**) derived from trastuzumab, by incubation with different BCN-linker-payload variants modified with maytansinoid (May) or duocarmycin SA (duo SA). First, for the non-cleavable linker, no notable difference in conjugation rate was noted between the constructs with (**3a**) or without (**3b**) the sulfamide moiety, leading in both cases to full conversion in 4 h. However, when the poorly water-soluble Val-Cit-PABC cleavable moiety was inserted into the linker, conjugation rates were in some cases very different between constructs with or without the HydraSpace™ moiety (Figure 2C): while the conjugation efficiency of the polar HydraSpace™ derivatives of BCN-linker-payload gave (near) complete conversion in 4 h for either maytansinoid or duocarmcyin payload, <20% conversion was noted for the PEG-only versions. An additional beneficial effect of the HydraSpace™ technology was noted for ADCs harboring the Val-Cit-PABC cleavable moiety in terms of a near two-fold reduction in time-dependent aggregation (Figure S4) of the maytansinoid-based ADCs containing carbamoyl sulfamide versus those derived containing PEG-only linker. Mitigation of aggregation propensity of ADCs is highly relevant since physical instability can compromise manufacturing, storage and in vivo performance, in particular for high DAR species [26], resulting in enhanced liver clearance [17], toxicity, and/or immunogenicity [27].

Next, the in vivo efficacy of the carbamoyl sulfamide moiety versus PEG-only spacer was assessed by evaluation in two different HER2-overexpressing mouse xenograft models (Figure 3 and Figure 4). For this study, we selected the trastuzumab-derived ADCs obtained by conjugation with **3a** or **3b**, both with a non-cleavable linker as in Kadcyla^®^. Thus, **3a** or **3b** were administered by tail vein injection to a PDX mouse grafted with HER2-overexpressing HBCx-13B (Figure 3A). In order to be able to assess a potential difference in efficacy, ADCs were administered at a sub-curative dose of 6 mg/kg only, since earlier studies showed full tumor regression in this model at 9 mg/kg [23]. It was found that treatment with PEG-only ADC **3b** showed tumor stasis, but HS-based **3a** led to (near) complete tumor regression with only partial tumor regrowth in a few mice. In light of the surprising difference in efficacy of ADCs purely based on difference in spacer technology, it was decided to corroborate this finding in another well-validated ADC xenograft model Karpas-299 with ADCs derived from brentuximab (anti-CD30) and payload monomethylauristatin E (MMAE). To this end, constructs **5a** (HydraSpace™) and **5b** (PEG-only) were synthesized from BCN and MMAE and conjugated to brentuximab-**1**. Efficacy of the resulting ADCs was assessed in vivo in Karpas-299 CDX mice grafted with Karpas-299 cell line (Figure 3B). Gratifyingly, the HydraSpace™-containing ADC (derived from **5a**) also in this case showed pronounced better tumor regression than the PEG-only ADC brentuximab-**5b** at the same dose (1 mg/kg), in particular during the first two weeks of the study.

Based on these observations, we were eager to find out whether the carbamoyl sulfamide technology would also be suitable for the generation of DAR4 ADCs by site-specific conjugation of a bivalent BCN-payload construct to glycan-remodeled antibody. Thus, we synthesized two branched BCN HydraSpace™ constructs (Figure 4A), one containing Ahx-maytansine (compound **6**) and one containing MMAE (compound **7**). In this case, it was noted that whereas one carbamoyl sulfamide unit suffices for the BCN-(MMAE)_2_ construct **7**, two sulfamide units were required in BCN-(maytansine)_2_ **6** in order to ensure solubility and conjugation efficiency. Thus, in the next step azidosugar-remodeled antibody **1**, derived from trastuzumab or brentuximab, was incubated with compound **6** or **7**, respectively, to give clean conversion to the desired DAR4 ADCs (trastuzumab-**6** and brentuximab-**7**).

Having the anti-HER2 and anti-CD30 ADCs in hand, the efficacy of each ADC was compared to comparison to the marketed drugs Kadcyla^®^ and Adcetris^®^, respectively, in the relevant mouse xenograft models. As becomes apparent, both glycan-conjugated ADCs containing HydraSpace™ showed full and durable tumor regression after a single administration of 9 mg/kg (for trastuzumab-**6**, Figure 4B) in the T226 model or 1 mg/kg (for brentuximab-**7**, Figure 4C) in the Karpas-299 model. In contrast, only partial response was noted in the same models for the marketed products Kadcyla^®^ and Adcetris^®^, respectively.

Tolerability was also determined in rats for all glycan-remodeled and marketed ADCs. In case of HER2-targeting trastuzumab-**6** and Kadcyla^®^, we noted an MTD for trastuzumab-**6** (~60 mg/kg), which is a modest increase with respect to ~50 mg/kg as experimentally determined for the marketed drug Kadcyla^®^ (reported MTD 46 mg/kg) [28]. Comparison of the CD30-targeting ADCs, however, showed a significant improvement in tolerability, increasing from ~15 mg/kg for Adcetris^®^ (reported MTD 18 mg/kg) [29] to ~60 mg/kg for brentuximab-**7**.

**Figure 5:**
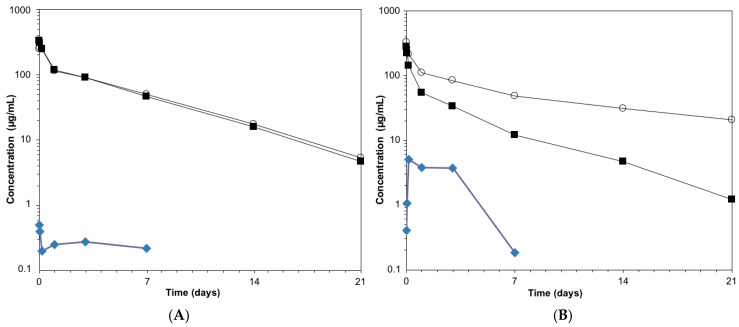
(**A**) PK profile of brentuximab-**7**; (**B**) PK profile of Adcetris^®^. Open circle = total antibody (by ELISA); solid square = total conjugated antibody (by ELISA); blue diamond = free MMAE (by LC-MS).

In order to provide a rationale for the observed increase in efficacy and safety for the GlycoConnect™/HydraSpace™ ADCs versus the marketed ADCs, in vivo pharmacokinetic profiles were determined by immunoassay (Figure 5). As illustrated for brentuximab-**7**, near complete overlap was noted for total mAb and total conjugated mAb (Figure 5A). In contrast, Adcetris^®^ showed significant premature release of payload (Figure 5B), attributed to the known poor stability of cysteine-maleimide conjugates [12,30]. We also studied pharmacokinetics of the DAR2 ADCs **5a** and **5b** (PEG-only), based on MMAE (Figure S11). Interestingly, we observed that the PK profile of **5a** (HydraSpace™) fully overlapped with that of the unmodified brentuximab, while a faster clearance was noted for ADC **5b** (PEG-only), which may be attributed to the higher hydrophobicity of the latter, leading to faster liver uptake. The pharmacokinetic comparison of ADCs based on maytansinoid payload (trastuzumab-**6** versus Kadcyla^®^) showed a similar picture (Figure S12) as for the MMAE-based ADCs.

## 5. Conclusions

With four marketed and >70 ADCs currently in the clinic, antibody–drug conjugates have emerged as promising drugs for targeted treatment of cancer. Nevertheless, the “magic bullet,” as formulated over 100 years ago by Ehrlich, is still elusive: ADCs are generally administered to patients at the maximum tolerated dose, which effectively means a therapeutic index of 1 (or less). A promising recent development is the emergence of second-generation ADCs with a better efficacy and tolerability profile, predominantly based on site-specific conjugation of toxic payload to engineered cysteine in the antibody [5]. An important shortcoming of the latter approach, however, is the fact that thiol–maleimide linkages often suffer from premature release of payload in circulation by retro-Michael reaction [5], leading to off-target toxicities, although careful selection of conjugation site [5,31] and/or the use of a self-hydrolysable maleimide variant [30] can significantly improve stability. Moreover, cysteine engineering of antibodies is, with a few exceptions [32,33], limited to a loading of two payloads per antibody (DAR2), which is preferable for highly potent drugs such as pyrrolobenzodiazepines (PBDs), but suboptimal for the broadly applied tubulin inhibitors such as maytansinoids (DM1, DM4) and auristatins (MMAE, MMAF). In addition, analysis of the ADC clinical pipeline indicates that the vast majority (>65%) of ADCs show an average drug-to-antibody ratio (DAR) of ~4.

We recently developed a non-genetic antibody conjugation technology based on enzymatic remodeling of the globally conserved glycan and copper-free click conjugation of payload, leading to highly stable ADCs. In fact, the site of attachment of the glycan (N297), in a cavity composed by the two heavy chains, seems to be of a “privileged” nature for the development of superior antibody–drug conjugates [30], as also underlined by the fact that multiple cysteine-engineered clinical ADCs are based on a S239C mutation, which resides in the same cavity. In order to further improve the versatility of the GlycoConnect™ technology in terms of conjugation efficiency of highly hydrophobic payloads, we were stimulated to screen for a more polar spacer technology. In this paper we show that introduction of an acylated sulfamide moiety next to traditional PEG spacers is highly favorable for the preparation and properties of antibody–drug conjugates. First, we established that acyl- and carbamoyl sulfamides (HydraSpace™ technology) display high polarity, most likely due to deprotonation of the central amino group at physiological pH. The resulting improved solubility allowed efficient conjugation of either monovalent or bivalent BCN payload constructs, thereby enabling tailoring of ADC format to DAR2 or DAR4, respectively. Remarkably, DAR2 ADCs based on HydraSpace™ were found to show improved in vivo efficacy versus the analogous “PEG-only” ADCs based on same antibody and payload. A possible explanation for this remarkable “HydraSpace™ effect” can be found in the almost identical PK profile with the naked, unconjugated antibody, whereas a faster clearance is noted for the ADC based on PEG-only as exemplified for brentuximab-**5a** versus brentuximab-**5b** (Figure S11). Consequently, it may be speculated that the superior efficacy of the HydraSpace™ ADC is correlated to higher exposure (AUC) with regard to the analogous ADC prepared with the traditional PEG-based spacer only, in line with data reported earlier on the correlation between linker polarity and PK [17].

Next, head-to-head evaluation in rodent models of two model DAR4 ADCs based on HydraSpace™ versus the marketed products Kadcyla^®^ and Adcetris^®^, as before based on the same antibody and payload components, indicated a remarkable increase in efficacy as well as in safety. Again, pharmacokinetic analysis indicated a clearly enhanced stability of the ADCs trastuzumab-**6** and brentuximab-**7** obtained by glycan remodeling/click chemistry versus the ADCs featuring thiol–maleimide linkages, as in the case in both Kadcyla^®^ and Adcetris^®^. Enhanced stability also provides a rationale for a higher MTD of the glycan-conjugated ADCs, which is most pronounced for brentuximab-**7** versus Adcetris^®^, in line with a recent report describing its instability in plasma [34].

Taken together, the carbamoyl sulfamide technology (HydraSpace™) described herein not only improves the manufacturability of ADCs through efficient conjugation of a toxic payload to an azido-modified monoclonal antibody [23], but also has the potential to provide next-generation ADCs with enhanced therapeutic index. Interestingly, metal-free click conjugation of branched payload constructs, most recently also applied for the preparation of a DAR4 ADCs by attachment of a branched azido-modified peptide carrying two auristatin or thailanstatin payloads, [35] has the potential to be extended to formats carrying two payloads with different mode-of-action, which may further boost the potential of the GlycoConnect™ technology in the generation of “dual warhead” ADCs [36,37,38]. Finally, we most recently corroborated that an ADC based on GlycoConnect and HydraSpace™ technologies also displays a significantly increased therapeutic index versus an ADC based on a cysteine-engineered antibody (unpublished data). Based on these observations, our current efforts are focused on CMC of the respective components (enzymes, UDP–azidosugar, and BCN–HydraSpace™) to enable the production of clinical grade GlycoConnect™ ADCs. With the first clinical studies commencing in 2018, it will be exciting to see the true value of GlycoConnect™ ADCs, featuring the HydraSpace™ technology, for targeted treatment of patients suffering from cancer.

## Supplementary Materials

The following are available online at http://www.mdpi.com/2073-4468/7/1/12/s1, Figure S1: General synthetic scheme for preparation of a carbamoyl sulfamide (**C**) from an alcohol (**A**) and an amine (**B**), Figure S2: HPLC retention times for HS compounds **3c** and **4a**, compared to those of PEG-only compounds **3d** and **4b**, respectively, Figure S3: Structures of model structures and HPLC retention times under acidic (0.1% TFA) or neutral conditions (buffer pH 7.4), Figure S4: Accelerated aggregation profile of a DAR2 trastuzumab ADC with Ahx-maytansine, based on either “PEG-only” spacer (**3d**) or “HS” (**3a**) under stressed conditions (pH 5, 40 °C), Figure S5: Aggregation study of trastuzumab-**6** versus Kadcyla^®^ in human serum at 37 °C shows negligible aggregation for trastuzumab-**6** and naked trastuzumab, while significant aggregation of Kadcyla^®^ is noted, Figure S6: Synthetic scheme for preparation of compounds **5a**, **5b**, and **7**, Figure S7: RP-HPLC traces of trastuzumab, trastuzumab-**1** and the trastuzumab ADC obtained by conjugation with **6**, Figure S8: Mass spectral analyses of IdeS-digested samples of trastuzumab, trastuzumab-**1**, and the trastuzumab ADC obtained by conjugation with **6**, Figure S9: RP-HPLC traces of brentuximab-**1** and the brentuximab ADCs obtained by conjugation with **5a**, **5b**, and **7**, Figure S10: Mass spectral analyses of IdeS-digested samples of brentuximab-**1**, brentuximab-**5a**, brentuximab-**5b**, and brentuximab-**7**, Figure S11: Pharmacokinetic profile of naked brentuximab or ADCs derived from brentuximab-**1** by conjugation with HydraSpace™ **5a** or with PEG only construct **5b**, Figure S12. (**A**) PK profile trastuzumab-**6** as determined by ELISA. (**B**) PK profile of Kadcyla^®^.
