# Correction: Verkade, J.M.M.; et al. A Polar Sulfamide Spacer Significantly Enhances the Manufacturability, Stability, and Therapeutic Index of Antibody–Drug Conjugates. *Antibodies* 2018, *7*, 12

**DOI:** 10.3390/antib7030034

**Published:** 2018-08-30

**Authors:** Jorge M. M. Verkade, Marloes A. Wijdeven, Remon van Geel, Brian M. G. Janssen, Sander S. van Berkel, Floris L. van Delft, Antibodies Editorial Office

**Affiliations:** 1 Synaffix BV, Industrielaan 63, 5349 AE Oss, The Netherlands; j.verkade@synaffix.com (J.M.M.V.); m.wijdeven@synaffix.com (M.A.W.); r.vangeel@synaffix.com (R.v.G.); b.janssen@synaffix.com (B.M.G.J.); s.vanberkel@synaffix.com (S.S.v.B.); f.vandelft@synaffix.com (F.L.v.D.); 2 MDPI, St. Alban-Anlage 66, 4052 Basel, Switzerland

The conflict of interest section of the published paper [1] has been updated as follows:

“All authors are employees of Synaffix BV, whose product HydraSpace was introduced in this paper.”

The manuscript will be updated and the original will remain online on the article webpage.
