# Supplementary material for: A Polar Sulfamide Spacer Significantly Enhances the Manufacturability, Stability, and Therapeutic Index of Antibody–Drug Conjugates
Source: Antibodies (Basel). 2018 Feb 20;7(1):12. doi: 10.3390/antib7010012 (PMC6698870; doi:10.3390/antib7010012)
Supplement: Supplementary file 1 [file antibodies-07-00012-s001.docx]

**Supplementary Material**

**Supplementary Figures and Table**

**Supplementary Figure 1**. General synthetic scheme for preparation of a carbamoyl sulfamide (**B**) from an alcohol (**A**) and an amine (**B**).


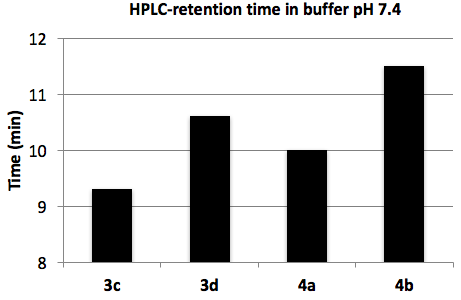


**Supplementary Figure 2**. HPLC-retention times for HS compounds **3c** and **4a**, compared to those of PEG-only compounds **3d** and **4b**, respectively.

**Supplementary Figure 3**. Structures of model structures and HPLC retention times under acidic (0.1% TFA) or neutral conditions (buffer pH 7.4). For compounds **D** (no sulfamide) and **H** (N-methylated sulfamide), no deprotonation can take place, hence no difference in retention time is observed. For monosulfamide compounds **E** and **F**, retention time difference is 2–2.4 min, which is further enhanced for the bis-sulfamide **G** (3.7 min).


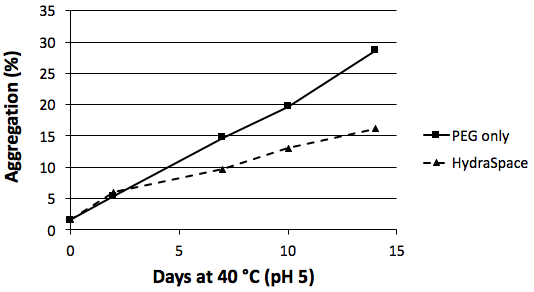


**Supplementary Figure 4**. Accelerated aggregation profile of a DAR2 trastuzumab ADC with Ahx-maytansine, based on either “PEG only” spacer (**3d**) or “HS” (**3a**) under stressed conditions (pH 5, 40 °C).

trastuzumab

Kadcyla

trastuzumab-**6**

**Supplementary Figure 5**. Aggregation study of trastuzumab-**6** versus Kadcyla in human serum at 37 °C shows negligible aggregation for trastuzumab-**6** and naked trastuzumab, while significant aggregation of Kadcyla is noted.

**Supplementary Figure 6**. Synthetic scheme for preparation of compounds **5a**, **5b** and **7**.


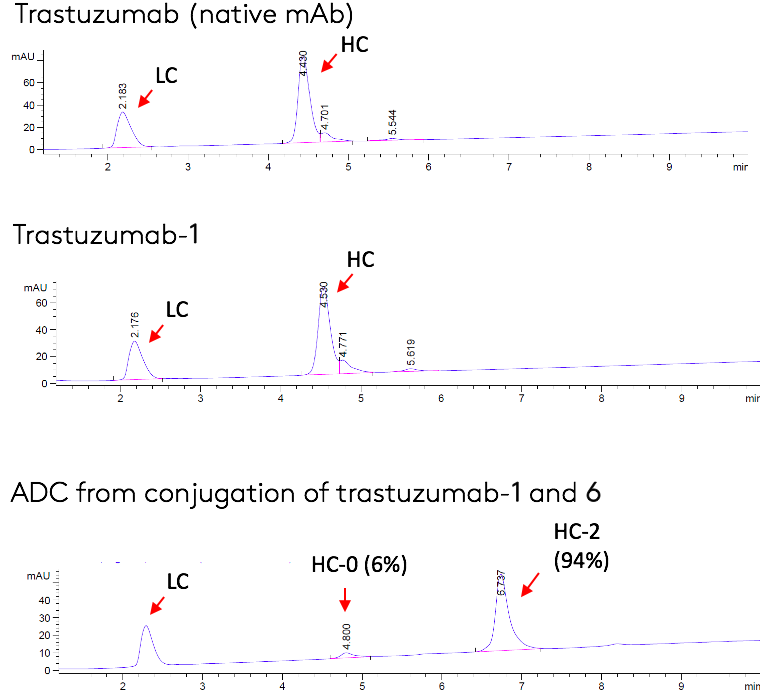


**Supplementary Figure 7**. RP-HPLC traces of trastuzumab, trastuzumab-**1** and the trastuzumab ADC obtained by conjugation with **6**. Based on peak area integration of the non-conjugated heavy chain (HC-0) and conjugated heavy chain (HC-2) the overall conversion from native mAb to GlycoConnect™ ADC was determined at 94%, corresponding to an average DAR of 3.78.


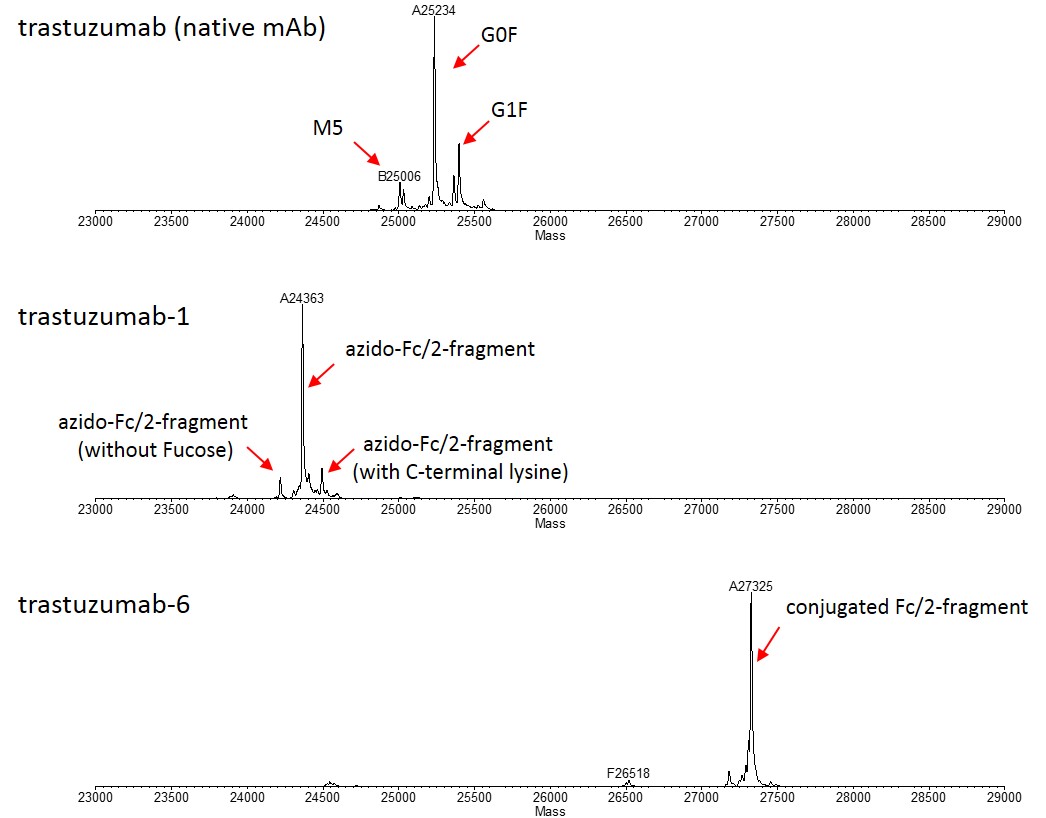


**Supplementary Figure 8**. Mass spectral analyses of IdeS-digested samples of trastuzumab, trastuzumab-**1** and the trastuzumab ADC obtained by conjugation with **6**.


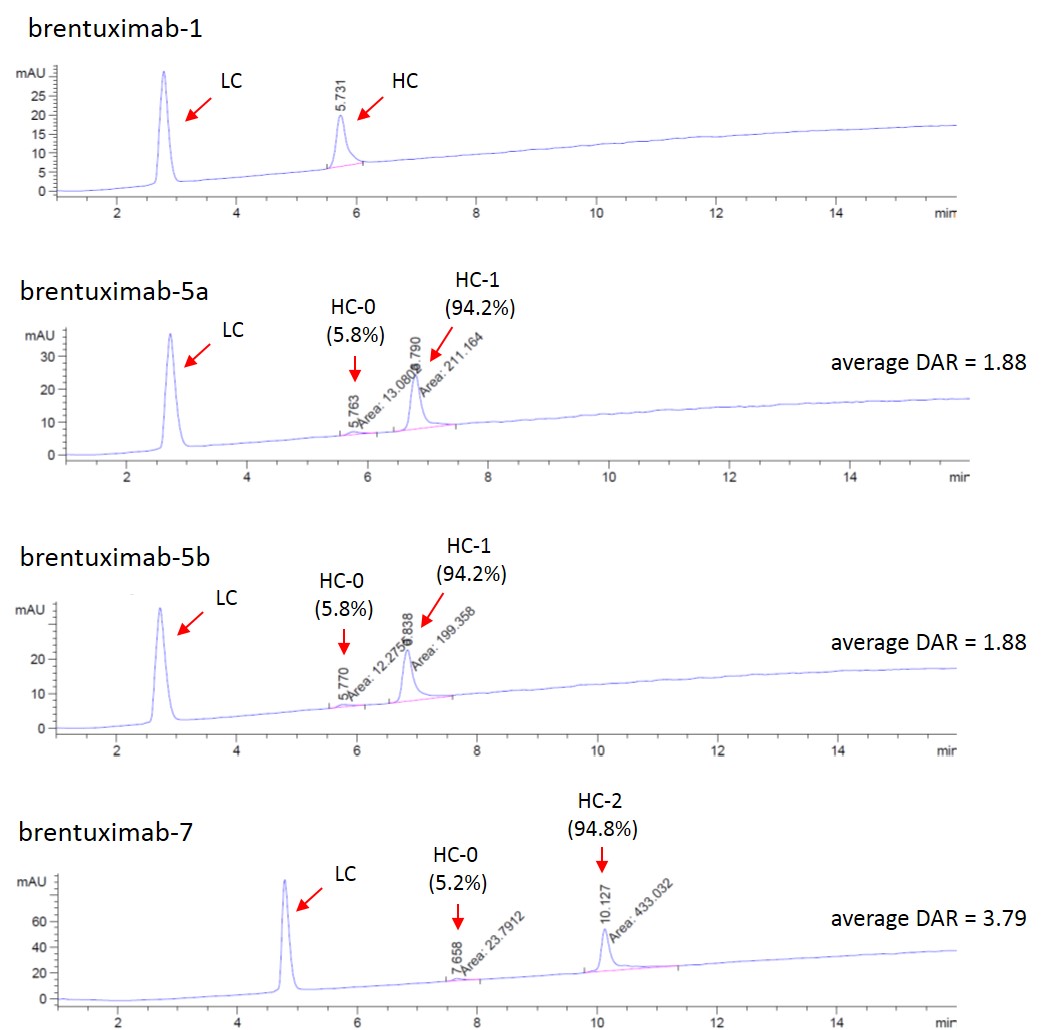


**Supplementary Figure 9**. RP-HPLC traces of brentuximab-**1** and the brentuximab ADCs obtained by conjugation with **5a**, **5b** and **7**. The average DAR is indicated for each ADC and was calculated based on the peak area of the non-conjugated heavy chain (HC-0) and conjugated heavy chain (HC-1 and HC-2).


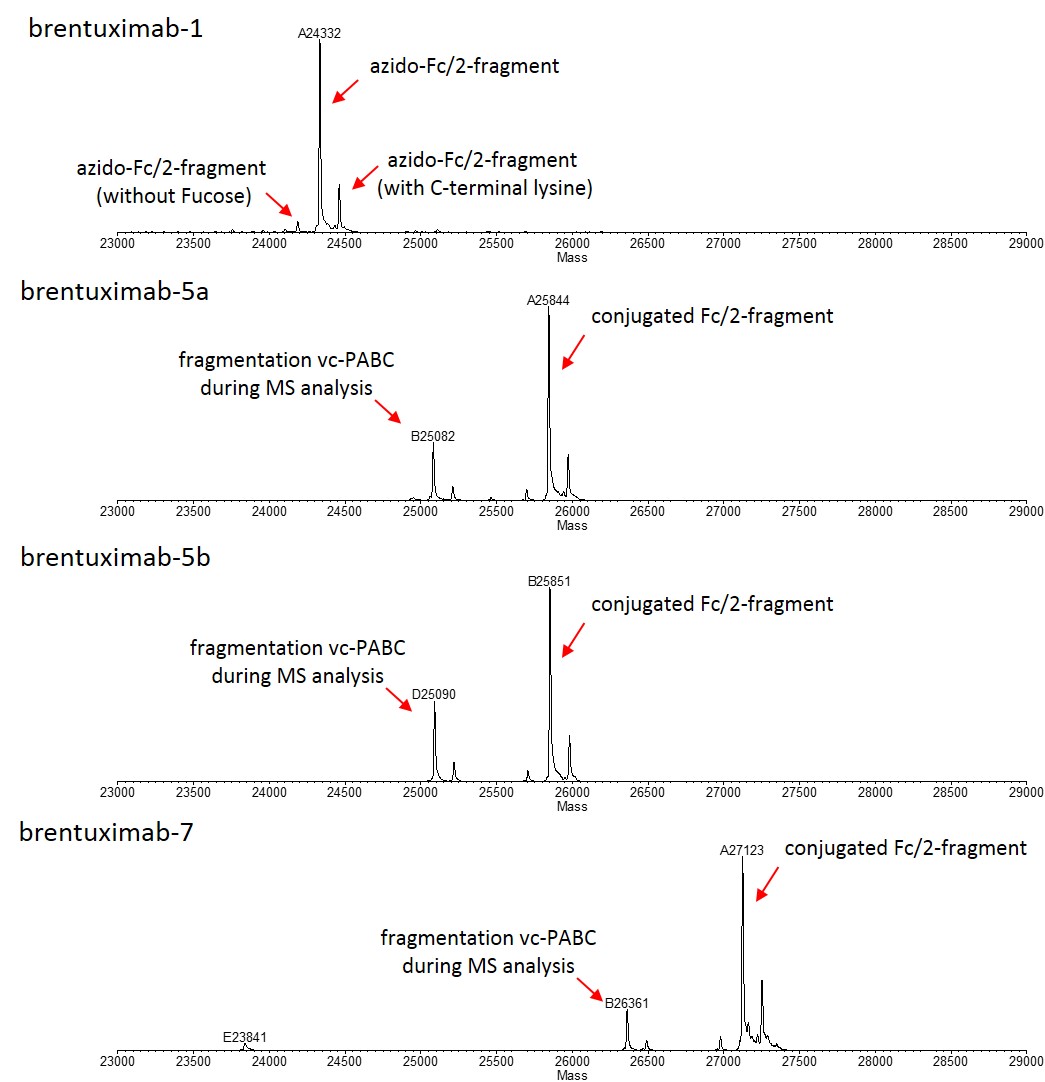


**Supplementary Figure 10**. Mass spectral analyses of IdeS-digested samples of brentuximab-**1**, brentuximab-**5a**, brentuximab-**5b** and brentuximab-**7**.


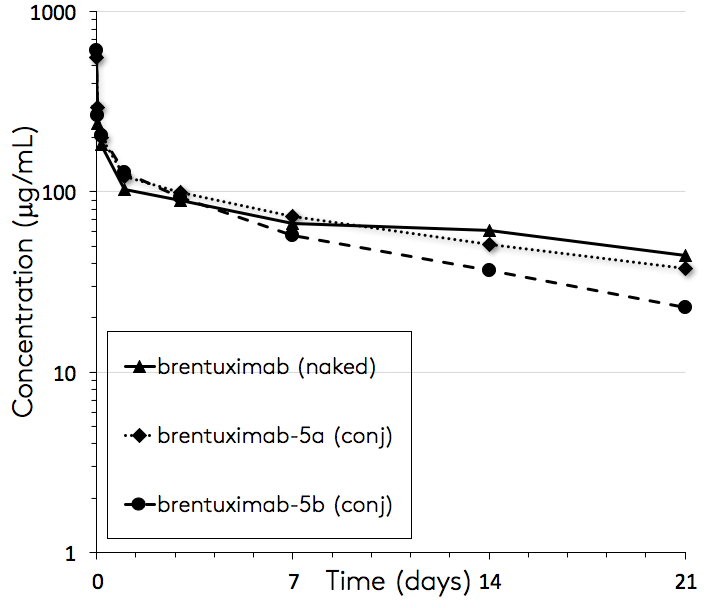


**Supplementary Figure 11**. Pharmacokinetic profile of naked brentuximab or ADCs derived from brentuximab-**1** by conjugation with HydraSpace™ **5a** or with PEG only construct **5b**. For clarity, only total conjugated ADC is depicted, which overlaps nearly completely with total antibody detected.

| **A**  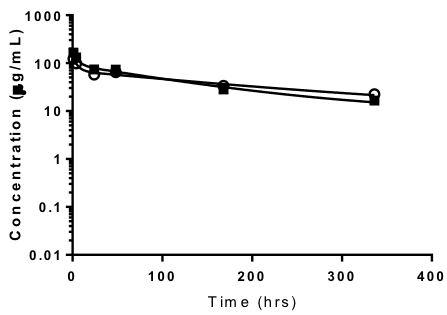 | **B**  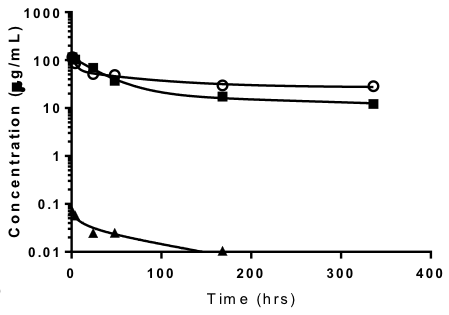 |
| --- | --- |

**Supplementary Figure 12**. (A) PK profile trastuzumab-**6** as determined by ELISA. (B) PK profile of Kadcyla®. Open circle: total antibody, closed square: total conjugated antibody. Free payload determination was performed by LC-MS (solid triangles), but was found to be below limit of detection of trastuzumab-**6**.

**Chemical Synthesis**

Unless noted otherwise, solvents were purchased from Sigma-Aldrich or Fisher Scientific and used as received. Thin layer chromatography was performed on silica gel-coated plates (Kieselgel 60 F254, Merck, Germany) with the indicated solvent mixture, spots were detected by KMnO_4_ staining (1.5 g KMnO_4_, 10 g K_2_CO_3_, 2.5 mL 5% NaOH-solution, 150 mL H_2_O), *p*-anisaldehyde staining (9.2 mL *p*-anisaldehyde, 321 mL EtOH, 17 mL H_2_O, 3.75 mL AcOH, 12.7 mL H_2_SO_4_), and UV-detection. NMR spectra were recorded on a Bruker Biospin 400 (400 MHz) and a Bruker DMX300 (300 MHz). Protein mass spectra (HRMS) were recorded on a JEOL AccuTOF JMS-T100CS (Electrospray Ionization (ESI) time-of-flight) or a JEOL AccuTOF JMS-100GCv (Electron Ionization (EI), Chemical Ionization (CI)). Low-resolution mass spectra (LRMS) were recorded on a ThermoScientific Advantage LCQ Linear ion-trap electrospray and a Waters LCMS consisting of a 2767 Sample manager, 2525 pump, 2996 UV-detector and a Micromass ZQ with an Xbridge™ C18 3.5 µm column (ESI). 9-Hydroxymethylbicyclo[6.1.0]non-4yne (BCN) alcohol and the BCN-OSu derivative thereof were prepared in-house. Chlorosulfonyl isocyanate was purchased from Sigma-Aldrich. Ahx-maytansinoid, Val-Cit-PABC-β-Ala-maytansinoid, Val-Cit-PABC-DMEDC-duocarmycin SA and Val-Cit-PABC-MMAE were purchased from Concortis (San Diego, USA).

**Synthesis of BCN-spacer-linker-payloads**

Compound **3a**. To a solution of BCN alcohol (0.15 g, 1.0 mmol) in DCM (15 mL) was added CSI (87 μL, 0.14 g, 1.0 mmol), Et_3_N (279 μL, 202 mg, 2.0 mmol) and a solution of H_2_N-PEG_3_-OH (251 mg, 1.3 mmol) in DCM (1 mL). After stirring for 2.5 h, the reaction mixture was quenched through addition of a solution of NH_4_Cl (sat., 20 mL). After separation, the aqueous layer was extracted with DCM (20 mL). The combined organic layers were dried (Na_2_SO_4_) and concentrated. The residue was purified by gradient column chromatography (0 → 10% MeOH in DCM). The product alcohol was obtained as colorless thick oil (254 mg, 0.57 mmol, 57%). ^1^H NMR (400 MHz, CDCl_3_) δ (ppm) 6.81 (br. s, 1H), 4.26 (d, *J* = 8.2 Hz, 2H), 3.80–3.70 (m, 4H), 3.70–3.58 (m, 10H), 3.36 (t, J = 4.7 Hz, 2H), 2.36–2.16 (m, 6H), 1.64–1.49 (m, 2H), 1.40 (quintet, *J* = 8.7 Hz, 1H), 1.04–0.92 (m, 2H).

To a solution of (1R,8S,9s)-bicyclo[6.1.0]non-4-yn-9-ylmethyl N-(2-(2-(2-(2-hydroxyethoxy)ethoxy)ethoxy)ethyl)sulfamoylcarbamate in DCM (10 mL) were added *p*-nitrophenyl chloroformate (38 mg, 0.187 mmol) and Et_3_N (79 µL, 57 mg, 0.56 mmol). After the mixture was allowed to stir for 1.5 h, water (10 mL) and DCM (10 mL) were added. After separation, the organic layer was dried (Na_2_SO_4_) and concentrated. 50 mg (44%) of the desired product was obtained after column chromatography (50% → 75% EtOAc in heptane). ^1^H NMR (400 MHz, CDCl_3_) δ (ppm) 8.31–8.25 (m, 2H), 7.43–7.38 (m, 2H), 4.49–4.44 (m, 2H), 4.27 (d, J = 8.2 Hz, 2H), 3.87–3.82 (m, 2H), 3.77–3.61 (m, 10H), 3.34 (t, J = 9.6 Hz, 2H), 2.36–2.16 (m, 6H), 1.75–1.45 (m, 2H), 1.39 (quintet , J = 8.7 Hz, 1H), 1.04–0.94 (m, 2H)

A solution of Ahx-maytansin (structure in **Fig. 2A**) as TFA salt (20 mg, 0.023 mmol) in DMF (2 mL) was added to a solution of the *p*-nitrophenyl carbonate obtained above (14 mg, 0.023 mmol) and Et_3_N (9.5 µL, 6.9 mg, 0.068 mmol) in DMF (2 mL) and the resulting reaction mixture was stirred for 24 h. Title compound **3a** was obtained in quantitative yield after silica gel column chromatography (29 mg, +99%). LCMS (ESI^+^) Calculated for C_58_H_84_ClN_6_O_18_ (M^+^-H_2_O) 1219.52, found 1219.84.

Compound **3b**. To solution of BCN-PEG_4_-OC(O)OSu (7.1 mg, 0.013 mmol) and Et_3_N (9.1 µL, 6.6 mg, 65.5 µmol) in 1 mL DMF was added to Ahx-maytansin.TFA (10 mg, 0.011 mmol). The reaction was stirred for 20 h at rt and subsequently concentrated under reduced pressure. The residue was purified via reversed phase (C18) HPLC chromatography (30 → 90% MeCN (1% AcOH) in H_2_O (1% AcOH). Product **3b** was obtained as colorless liquid (8.9 mg, 7.5 µmol, 68%). LRMS (ESI^+^) m/z calcd for C_60_H_87_ClN_5_O_16_ (M^+^-H_2_O) = 1168.58; found 1168.87.

Compound **3c**. To a solution of BCN alcohol (1.5 g, 10 mmol) in DCM (150 mL), under a N_2_ atmosphere, was added CSI (0.87 mL, 1.4 g, 10 mmol), Et_3_N (2.8 mL, 2.0 g, 20 mmol) and 2-(2-aminoethoxy)ethanol (1.2 mL, 1.26 g, 12 mmol). The mixture was stirred for 10 min and quenched through addition of aqueous NH_4_Cl (sat., 150 mL). After separation, the aqueous layers was extracted with DCM (150 mL). The combined organic layers were dried (Na_2_SO_4_) and concentrated. The residue was purified with column chromatography. Product alcohol was obtained as slightly yellow thick oil (2.06 g, 5.72 mmol, 57%). ^1^H NMR (400 MHz, CDCl_3_) δ (ppm) 6.0 (bs, 1H), 4.28 (d, *J* = 8.2 Hz, 2H), 3.78–3.73 (m, 2H), 3.66–3.61 (m, 2H), 3.61–3.55 (m, 2H), 3.34 (t, J = 4.9 Hz, 2H), 2.37­–2.15 (m, 6H), 1.64–1.48 (m, 2H), 1.40 (quintet, *J* = 8.7 Hz, 1H), 1.05–0.92 (m, 2H).

To a solution of the product alcohol (229 mg, 0.64 mmol) in DCM (20 mL) were added *p*-nitrophenyl chloroformate (128 mg, 0.64 mmol) and Et_3_N (268 µL, 194 mg, 1.92 mmol). The mixture was stirred overnight at rt and subsequently concentrated under reduced pressure. The residue was purified via gradient column chromatography (20 → 70% EtOAc in heptane (1% AcOH) to afford the desired activated carbonate as a white solid (206 mg, 0.39 mmol, 61%). ^1^H NMR (400 MHz, CDCl_3_) δ (ppm) 8.31–8.26 (m, 2H), 7.45–7.40 (m, 2H), 5.56 (t, *J* = 6.0 Hz, 1H), 4.48–4.40 (m, 2H), 4.27 (d, *J* = 8.2 Hz, 2H), 3.81–3.75 (m, 2H), 3.68 (t, *J* = 5.0 Hz, 2H), 3.38–3.30 (m, 2H), 2.36–2.14 (m, 6H), 1.61–1.45 (m, 2H), 1.38 (quintet, *J* = 8.7 Hz, 1H), 1.04–0.94 (m, 2H).

To a solution of the activated carbonate (4.1 mg, 7.8 µmol) and Et_3_N (3.3 µL, 2.4 mg, 23.4 µmol) in DMF (1 mL) was added a solution of H-Val-Cit-PABC-Ahx-maytansin (10 mg, 8.6 µmol) in DMF (100 µL). After 20 h, 2,2′-(ethylenedioxy)bis(ethylamine) (5.7 µL, 5.6 mg, 38 µmol) was added and the mixture was concentrated under reduced pressure. The residue was purified via reversed phase (C18) HPLC chromatography (30 → 90% MeCN (1% AcOH) in H_2_O (1% AcOH) to give **3c** (2.2 mg, 1.4 µmol, 18%). LRMS (ESI^+^) m/z calcd for C_73_H_103_ClN_11_O_21_S (M-18+H^+^) = 1536.67; found 1537.08.

Compound **3d**. To a suspension of amino-dPEG_4_-acid (0.123 g, 0.46 mmol) in DMF (3mL), were subsequently added BCN-OSu (0.102 g, 0.35 mmol) and triethylamine (0.16 mL, 1.15 mmol). The reaction mixture was stirred for 3h at rt, after which EDCI.HCl (0.088 g, 0.46 mmol) and NHS (8.8 mg, 0.076 mmol) were added. The resulting solution was stirred for 17 h at rt and poured into 10 mL NaHCO_3_ (sat.) and 15 mL EtOAc. The layers were separated and the organic phase was washed with sat. NaHCO_3_ (9 mL) and H_2_O (7.5 mL). The organic phase was dried (Na_2_SO_4_), filtered and concentrated *in vacuo*. Gradient flash chromatography (MeCN → MeCN:H_2_O 30:1) afforded the activated ester as a colorless oil (50 mg, 0.093 mmol, 27%). ^1^HNMR (400 MHz, CDCl_3_): δ 5.26 (bs, 1H), 4.14 (d, *J* = 8.0 Hz, 2H), 3.84 (t, *J* = 6.5 Hz, 2H), 3.70-3.59 (m, 12 H), 3.56 (t, *J* = 5.1 Hz, 2H), 3.41–3.33 (m, 2H), 2.90 (t, *J* = 6.5 Hz, 2H), 2.88–2.78 (m, 4H), 2.37-2.15 (m, 6H), 1.69-1.51 (m, 2H), 1.36 (quintet, *J* = 8.7 Hz, 1H), 1.00-0.89 (m, 2H). LRMS (ESI+) calcd for C_26_H_38_N_2_O_10_Na^+^ (M+Na^+^) 561.24, found 561.01.

Next, to a solution of Val-Cit-PABC-Ahx-maytansine (30 mg, 23 µmol) in MeCN (6 mL)/DMF (2 mL)/Et_3_N (16 µL) was added to a solution of BCN-dPEG_4_-C(O)OSu as prepared above (13.4 mg, 24.9 µmol) in MeCN (2 mL). The mixture was left for 22 h and concentrated. The crude residue was purified by silica column chromatography (EtOAc to 50% MeOH in EtOAc). The desired product **3d** was obtained as a thick colorless oil (28 mg, 18 µmol, 72%). LRMS (ESI^+^) m/z calcd for C_79_H_116_ClN_10_O_22_^+^ (M+H^+^) = 1591.79; found 1592.94.

Compound **4a**. A solution of BCN-sulfamide-PEG_3_-*p*-nitrophenyl carbonate (6.0 mg, 9.8 µmol) and Et_3_N (6.8 µL, 4.9 mg, 48.5 µmol) in DMF (1 mL) was added to H-Val-Cit-PABC-DMEDC-duocarmycin SA (10 mg, 0.0097 mmol). After 22 h, 2,2′-(ethylenedioxy)bis(ethylamine) (2.8 µL, 2.8 mg, 19 µmol) was added. After 1h, the reaction mixture was concentrated under reduced pressure and the residue was purified via reversed phase (C18) HPLC chromatography (30 → 90% MeCN (1% AcOH) in H_2_O (1% AcOH). Product **4a** was obtained as a white solid (6.4 mg, 4.2 µmol. 44%). LRMS (ESI^+^) m/z calcd for C_69_H_92_ClN_12_O_22_S (M+H^+^) = 1507.59; found 1508.00.

Compound **4b**. A solution of BCN-PEG_4_-OC(O)OSu (6.6 mg, 0.012 mmol) and Et_3_N (6.8 µL, 4.9 mg, 48.5 µmol) in 1 mL DMF was added to Val-Cit-PABC-derivative of DMEDC-duocarmycin SA (10 mg, 0.0097 mmol) (structure in **Fig. 2A**). After 18 h, 2,2′-(ethylenedioxy)bis(ethylamine) (1.8 µL, 1.8 mg, 12 µmol) was added and the mixture was concentrated under reduced pressure. The residue was purified via reversed phase (C18) HPLC chromatography (30 → 90% MeCN (1% AcOH) in H_2_O (1% AcOH). Product **4b** was obtained as a colorless film (7.5 mg, 5.1 µmol, 53%). LRMS (ESI^+^) m/z calcd for C_71_H_94_ClN_10_O_21_ (M+H^+^) = 1457.63; found 1456.89.

Compound **5a** (prepared according to Suppl. **Fig. 5**). To a solution of BCN alcohol (1.5 g, 10 mmol) in DCM (150 mL), under a N_2_ atmosphere, was added CSI (0.87 mL, 1.4 g, 10 mmol), Et_3_N (2.8 mL, 2.0 g, 20 mmol) and 2-(2-aminoethoxy)ethanol (1.2 mL, 1.26 g, 12 mmol). The mixture was stirred for 10 min and quenched through addition of aqueous NH_4_Cl (sat., 150 mL). After separation, the aqueous layers was extracted with DCM (150 mL). The combined organic layers were dried (Na_2_SO_4_) and concentrated. The residue was purified with column chromatography. The product alcohol was obtained as slightly yellow thick oil (2.06 g, 5.72 mmol, 57%). ^1^H NMR (400 MHz, CDCl_3_) δ (ppm) 6.0 (bs, 1H), 4.28 (d, *J* = 8.2 Hz, 2H), 3.78–3.73 (m, 2H), 3.66–3.61 (m, 2H), 3.61–3.55 (m, 2H), 3.34 (t, J = 4.9 Hz, 2H), 2.37­–2.15 (m, 6H), 1.64–1.48 (m, 2H), 1.40 (quintet, *J* = 8.7 Hz, 1H), 1.05–0.92 (m, 2H).

To a solution of the alcohol prepared above (229 mg, 0.64 mmol) in DCM (20 mL) were added *p*-nitrophenyl chloroformate (128 mg, 0.64 mmol) and Et_3_N (268 µL, 194 mg, 1.92 mmol). The mixture was stirred overnight at rt and subsequently concentrated under reduced pressure. The residue was purified via gradient column chromatography (20 → 70% EtOAc in heptane (1% AcOH) to afford the PNP carbonate derivative as a white solid (206 mg, 0.39 mmol, 61%). ^1^H NMR (400 MHz, CDCl_3_) δ (ppm) 8.31–8.26 (m, 2H), 7.45–7.40 (m, 2H), 5.56 (t, *J* = 6.0 Hz, 1H), 4.48–4.40 (m, 2H), 4.27 (d, *J* = 8.2 Hz, 2H), 3.81–3.75 (m, 2H), 3.68 (t, *J* = 5.0 Hz, 2H), 3.38–3.30 (m, 2H), 2.36–2.14 (m, 6H), 1.61–1.45 (m, 2H), 1.38 (quintet, *J* = 8.7 Hz, 1H), 1.04–0.94 (m, 2H).

To a solution of the PNP carbonate prepared above (4.7 mg, 9.0 µmol) in DMF (200 µL) was added solid H-Val-Cit-PABC-MMAE (vc-PABC-MMAE, 10 mg, 8.1 µmol) followed by addition of Et_3_N (3.7 µL, 2.7 mg, 27 µmol). After 23 h, 2′-(ethylenedioxy)bis(ethylamine) (1.3 µL, 1.3 mg, 8.9 µmol) in DMF was added (13 µL of 10% solution in DMF). The mixture was left for 4h and purified *via* reversed phase (C18) HPLC chromatography (30→90% MeCN (1% AcOH) in H_2_O (1 % AcOH). The product **5a** was obtained as a colourless film (10.7 mg, 7.1 µmol, 87%) LCMS (ESI^+^) calculated for C_74_H_117_N_12_O_19_S^+^ (M+H^+^) 1509.83 found 1510.59.

Compound **5b** (prepared according to Suppl. **Fig. 5**). To a solution of 2-(2-(2-(2-aminoethoxy)ethoxy)ethoxy)ethanol (539 mg, 2.79 mmol) in DCM (100 mL) were added BCN-OSu carbonate (0.74 g, 2.54 mmol) and Et_3_N (1.06 mL, 771 mg, 7.62 mmol). The resulting solution was stirred for 2.5 h and washed with a saturated aqueous solution of NH_4_Cl (100 mL). After separation, the aqueous phase was extracted with DCM (100 mL). The combined organic phases were dried (Na_2_SO_4_) and concentrated. The residue was purified with column chromatography (MeOH in DCM 0 → 10%). The product alcohol was obtained as a colourless oil (965 mg, 2.61 mmol, quant). ^1^H NMR (400 MHz, CDCl_3_) δ (ppm) 5.93 (bs, 1H), 4.14 (d, *J* = 8.0 Hz, 2H), 3.77–3.69 (m, 4H), 3.68–3.59 (m, 8H), 3.58–3.52 (m, 2H), 3.42–3.32 (m, 2H), 2.35–2.16 (m, 6H), 1.66–1.51 (m, 2H), 1.36 (quintet, *J* = 8.7 Hz, 1H), 0.99–0.87 (m, 2H).

To a solution of the alcohol prepared above (0.96 g, 2.59 mmol) in DCM (50 mL) was added *p*-nitrophenyl chloroformate (680 mg, 3.37 mmol) and Et_3_N (1.08 mL, 784 mg, 7.75 mmol). The mixture was stirred for 16 h and concentrated. The residue was purified twice with column chromatography (20% → 70% EtOAc in heptane (column 1) and 20% → 100% EtOAc in heptane (column 2)). The product PNP carbonate was obtained as a slightly yellow thick oil (0.91 g, 1.70 mmol, 66%). ^1^H NMR (400 MHz, CDCl_3_) δ (ppm) 8.31–8.26 (m, 2H), 7.42–7.37 (m, 2H), 5.19 (bs, 1H), 4.47–4.43 (m, 2H), 4.15 (d, *J* = 8.0 Hz, 2H), 3.84–3.80 (m, 2H), 3.74–3.61 (m, 8H), 3.59–3.53 (m, 2H), 3.42–3.32 (m, 2H), 2.35–2.16 (m, 6H), 1.66–1.50 (m, 2H), 1.40–1.30 (m, 1H), 1.00–0.85 (m, 2H).

To a solution of H-Val-Cit-PABC-MMAE (vc-PABC-MMAE; 13.9 mg; 0.011 mmol in DMF (400 µL) were added Et_3_N (3.4 µL, 2.5 mg, 24.3 µmol) and a solution of the PNP carbonate prepared above (3.0 mg, 5.6 µmol) in DMF (200 µL). After 25 min, additional Et_3_N (1.1 µL, 0.80 mg, 7.9 µmol) and BCN-PEG_4_-OPNP (**110**, 2.2 mg, 4.1 µmol in DMF (33 µL)) were added. After 17.5 h, 2′-(ethylenedioxy)bis(ethylamine) (1.2 µL, 1.2 mg, 8.1 µmol) in DMF was added (12 µL of 10% solution in DMF). The mixture was left over night in the freezer and purified *via* reversed phase (C18) HPLC chromatography (30→90% MeCN (1% AcOH) in H_2_O (1 % AcOH). The product **5b** was obtained as a colourless film (10.9 mg, 7.2 µmol. 74%) LCMS (ESI^+^) calculated for C_78_H_124_N_11_O_19_^+^ (M+H^+^) 1518.91 found 1519.09.

Compound **6**. To a solution of BCN alcohol (1.5 g, 10 mmol) in DCM (150 mL), under a N_2_ atmosphere, was added CSI (0.87 mL, 1.4 g, 10 mmol), Et_3_N (2.8 mL, 2.0 g, 20 mmol) and 2”-(2’-aminoethoxy)ethanol (1.2 mL, 1.26 g, 12 mmol). The mixture was stirred for 10 min and quenched through addition of aqueous NH_4_Cl (sat., 150 mL). After separation, the aqueous layers was extracted with DCM (150 mL). The combined organic layers were dried (Na_2_SO_4_) and concentrated. The residue was purified with column chromatography. Product BCN-sulfamide alcohol was obtained as slightly yellow thick oil (2.06 g, 5.72 mmol, 57%). ^1^H NMR (400 MHz, CDCl_3_) δ (ppm) 6.0 (bs, 1H), 4.28 (d, *J* = 8.2 Hz, 2H), 3.78–3.73 (m, 2H), 3.66–3.61 (m, 2H), 3.61–3.55 (m, 2H), 3.34 (t, J = 4.9 Hz, 2H), 2.37­–2.15 (m, 6H), 1.64–1.48 (m, 2H), 1.40 (quintet, *J* = 8.7 Hz, 1H), 1.05–0.92 (m, 2H).

To a stirring solution of the BCN-sulfamide alcohol obtained above (47 mg, 0.13 mmol) in DCM (10 mL) was added CSI (11 µL, 18 mg, 0.13 mmol). After 30 min, Et_3_N (91 µL, 66 mg, 0.65 mmol) and a solution of diethanolamine (16 mg, 0.16 mmol) in DMF (0.5 mL) were added. After 30 minutes *p*-nitrophenyl chloroformate (52 mg, 0.26 mmol) and Et_3_N (54 µL, 39 mg, 0.39 mmol) were added. After an additional 4.5 h, the reaction mixture was concentrated and the residue was purified by gradient column chromatography (33 → 66% EtOAc/heptane (1% AcOH)) to afford intermediate activated carbonate as a colorless oil (88 mg, 0.098 mmol, 75%). ^1^H NMR (400 MHz, CDCl_3_) δ (ppm) 8.28­–8.23 (m, 4H), 7.42­–7.35 (m, 4H), 5.84 (bs, 1H), 4.52 (t, *J* = 5.4 Hz, 4H), 4.30 (d, *J* = 8.3 Hz, 2H), 4.27­–4.22 (m, 2H), 3.86 (t, *J* = 5.3 Hz, 4H), 3.69–3.65 (m, 2H), 3.64–3.59 (m, 2H), 3.30–3.22 (m, 2H), 2.34–2.14 (m, 6H), 1.62–1.46 (m, 2H), 1.38 (quintet, *J* = 8.7 Hz, 1H), 1.04–0.92 (m, 2H).

A solution of the intermediate diol obtained above (3.9 mg, 4.3 µmol) and Et_3_N (3.0 µL, 2.2 mg, 21.5 µmol) in DMF (1 mL) was added to a solution of H-Val-Cit-PABC-Ahx-maytansin (10 mg, 8.6 µmol) in DMF (100 µL). The mixture was allowed to react o.n. and concentrated. The residue was purified via reversed phase (C18) HPLC chromatography (30 → 90% MeCN (1% AcOH) in H_2_O (1% AcOH) to give product **6** (3.9 mg, 1.32 µmol, 31%). LRMS (ESI^+^) m/z calcd for C_136_H_196_Cl_2_N_22_O_43_S_2_ (M+2H^+^)/2 =1480.13; found 1480.35. As a side-product, the mono-Ahx-May substituted derivative of **6** was isolated (not depicted). LRMS (ESI+) calculated for C_85_H_119_ClN_14_O_31_S_2_ ^2+^ m/z 965.36, found 965.54.

Compound **7** (prepared according to Suppl. **Fig. 5**). To solution of the PNP carbonate prepared in the synthesis of **5a** (0.39 g; 0.734 mmol) in DCM (30 mL) were added a solution of diethanolamine (DEA, 107 mg; 1.02 mmol) in DMF (2 mL) and Et_3_N (305 µL; 221 mg; 2.19 mmol). The resulting mixture was stirred at rt for 17 h and washed with a saturated aqueous solution of NH_4_Cl (30 mL). The aqueous phase was extracted with DCM (30 mL) and the combined organic layers were dried (Na_2_SO_4_) and concentrated. The residue was purified by flash column chromatography (DCM → MeOH/DCM 1/9). The product diol was obtained as a colourless film (163 mg; 0.33 mmol; 45%). ^1^H NMR (400 MHz, CDCl_3_) δ (ppm) 6.29 (bs, 1H), 4.33­–4.29 (m, 2H), 4.28 (d, *J* = 8.2 Hz, 2H), 3.90–3.80 (m, 4H), 3.69–3.64 (m, 2H), 3.61 (t, *J* = 4.8 Hz, 2H), 3.52 (t, *J* = 5.0 Hz, 4H), 3.32 (t, *J* = 5.1 Hz, 2H), 2.37–2.18 (m, 6H), 1.60–1.55 (m, 2H), 1.39 (quintet, J = 8.7 Hz, 1H), 1.05–0.94 (m, 2H).

To a solution of the diol prepared above (163 mg, 0.33 mmol) and 4-nitrophenyl chloroformate (134 mg, 0.66 mmol) in DCM (10 mL) was added Et_3_N (230 µL; 167 mg; 1.65 mmol). The reaction mixture was stirred for 17 h and concentrated. The residue was purified by flash column chromatography (50% EtOAc in heptane → 100% EtOAc). The product was obtained as a colourless oil (69 mg; 0.084 mmol; 25%). ^1^H NMR (400 MHz, CDCl_3_) δ (ppm) 8.29–8.23 (m, 4H), 7.42–7.35 (m, 4H), 5.81–5.71 (m, 1H), 4.53–4.43 (m, 4H), 4.36–4.30 (m, 2H), 4.25 (d, J = 8.2 Hz, 2H), 3.81–3.70 (m, 4H), 3.70–3.65 (m, 2H), 3.62–3.56 (m, 2H), 3.32–3.24 (m, 2H), 2.34–2.14 (m, 6H), 1.60–1.45 (m, 2H), 1.35 (quintet, *J* = 8.7 Hz, 1H), 1.02–0.91 (m, 2H).

To a solution of bis PNP-carbonate (27 mg, 33 µmol) in DMF (400 µL) were added triethylamine (22 µl; 16 mg; 158 µmol) and a solution of vc-PABC-MMAE.TFA (96 mg; 78 µmol) in DMF (1.0 mL). The mixture was left standing for 19 h and 2,2′-(ethylenedioxy)bis(ethylamine) (37 µL, 38 mg, 253 µmol) was added. After 2 h, the reaction mixture was diluted with DMF (100 µL) and purified by RP HPLC (C18, 30% → 90% MeCN (1% AcOH) in water (1% AcOH). The desired product **7** was obtained as a colourless film (41 mg, 14.7 µmol, 45%). LCMS (ESI^+^) calculated for C_138_H_219_N_23_O_35_S^2+^ (M+2H^+^) 1395.79 found 1396.31.

**Synthesis of compounds D–H**

Structures of compounds **D**–**H** provided in Suppl. **Fig. 3**.

Compound **D**. To a mixture of BCN-OSu carbonate (291 mg, 1.00 mmol) in DCM (25 mL) were added 7-aminoheptanoic acid and Et_3_N (417 μl, 303 mg, 3.00 mmol) and DMF (10 mL) was added. After evaporation (40 °C) of DCM, the resulting mixture was stirred for 10 min and an aqueous solution (0.1 M) of NaHCO_3_ was added. After the reaction mixture was stirred for an additional 3 h, it was poured out in saturated aqueous NH_4_Cl (50 mL) and extracted with DCM (2 × 50 mL). The combined organic layers were dried (Na_2_SO_4_) and concentrated. The residue was taken up in DCM (25 mL), *N*-​(3-​dimethylaminopropyl)​-*N*′-ethylcarbodiimide hydrochloride (EDCI.HCl, 249 mg, 1.30 mmol) and *N-­*hydroxysuccinimide (150, 1.30 mmol) were added and the resulting mixture was stirred for 18 h. After addition of water (50 mL), the layers were separated and the aqueous phase was extracted with DCM (2 x 25 mL). The combined organic layers were washed with brine (50 mL), dried (Na_2_SO_4_) and concentrated. Column chromatography yielded the intermediate NHS ester derivative as a colorless thick oil (233 mg, 0.56 mmol, 56%). ^1^H NMR (400 MHz, CDCl_3_) δ (ppm) 4.69 (s, 1H), 4.14 (d, *J* = 8.0 Hz, 2H), 3.17 (dd, *J* = 13.6, 6.9 Hz, 2H), 2.91–2.77 (m, 4H), 2.61 (t, *J* = 7.4 Hz, 2H), 2.37–2.12 (m, 6H), 1.83–1.69 (m, 2H), 1.66–1.17 (m, 9H), 1.01–0.90 (m, 2H). Next, to a solution of the intermediate BCN-aminoheptanoic acid NHS ester (49 mg, 0.12 mmol) in DCM (12 mL) were added benzylamine (19 μL, 19 mg, 0.18 mmol) and Et_3_N (50 μL, 36 mg, 0.36 mmol). The mixture was stirred for 19 h and DCM (10 mL) and saturated aqueous NH_4_Cl (20 mL) were added. The organic layer was dried (Na_2_SO_4_) and concentrated. After gradient column chromatography (25% → 50% EtOAc in heptane) compound **D** was obtained as a white solid (31 mg, 0.076 mmol, 63%). ^1^H NMR (400 MHz, CDCl_3_) δ (ppm) 7.38–7.27 (m, 5H), 5.72 (bs, 1H), 4.64 (bs, 1H), 4.45 (d, *J* = 5.6 Hz, 2H), 4.13 (d, *J* = 8.1 Hz, 2H), 3.16 (dd, *J* = 12.8, 6.3 Hz, 2H), 2.38–2.13 (m, 8H), 1.75–1.11 (m, 11H), 1.00–0.88 (m, 2H).

Compound **E.** Under an atmosphere of N_2_, to a cooled solution (–78 °C) of *tert*-butanol in Et_2_O (20 mL) was added chlorosulfonyl isocyanate (CSI) and the mixture was allowed to reach rt. After 45 min, the mixture was concentrated and the resulting *tert*-butyl chlorosulfonylcarbamate was used in the next step without further purification (considered 68% pure). Thus, to a solution of the crude *tert*-butyl chlorosulfonylcarbamate (199 mg crude = 135 mg, 0.63 mmol) in DCM (10 mL) was added Et_3_N (263 μL, 191 mg, 1.89 mmol) and benzylamine (82 μL, 81 mg, 0.85 mmol). The mixture was stirred for 2 h and quenched with saturated aqueous NH_4_Cl. DCM (10 mL) was added and the layers were separated. The organic layer was dried (Na_2_SO_4_) and concentrated. After gradient column chromatography (25% → 50% EtOAc in heptane), the *tert*-butyl *N*-benzylsulfamoylcarbamate was obtained as a white solid (169 mg, 0.59 mmol). ^1^H NMR (400 MHz, CDCl_3_) δ (ppm) 7.41–7.29 (m, 5H), 7.03 (bs, 1H) 5.41–5.30 (m, 1H), 4.30–4.20 (m, 2H), 1.46 (s, 9H).

To a solution of *tert*-butyl *N*-benzylsulfamoylcarbamate (108 mg, 0.38) in DCM (10 mL) was added trifluoroacetic acid (2 mL). The reaction mixture was stirred for 1.5 h and poured into saturated aqueous NaHCO_3_ (50 mL). After addition of another 50 mL of saturated aqueous NaHCO_3_, the aqueous mixture was extracted with DCM (50 mL). The organic layer was dried (Na_2_SO_4_) and concentrated. The *N-*benzyl sulfamide was obtained as a white solid (36 mg, 0.19 mmol, 50%). ^1^H NMR (400 MHz, CDCl_3_) δ (ppm) 7.41–7.30 (m, 5H), 4.32 (d, *J* = 6.1 Hz, 2H).

To a solution of BCN-OSu carbonate in MeCN (5 mL) were added 7-aminoheptanoic acid (145 mg, 1.0 mmol) in 0.1 M aqueous NaHCO3 (30 mL) and MeCN (25 mL). The mixture was stirred for 4 h and partially concentrated. Aqueous saturated NH_4_Cl (30 mL) was added and after extraction with DCM (2 × 30 mL), the combined organics were dried (Na_2_SO_4_) and concentrated. The product was used in the step without purification. ^1^H NMR (400 MHz, CDCl_3_) δ (ppm) 4.68 (bs, 1H), 4.14 (d, *J* = 7.9 Hz, 2H), 3.17 (dd, *J* = 12.8, 6.3 Hz, 2H), 2.35 (t, *J* = 7.5 Hz, 2H), 2.32–2.09 (m, 6H), 1.70–1.25 (m, 11H), 0.94 (t, *J* = 9.7 Hz, 2H). To a solution of BCN-aminoheptanoic acid (44 mg, 0.136 mmol) in DCM (5 mL) were added EDCI.HCl (39 mg, 0.204 mmol), DMAP (2.9 mg, 0.024 mmol) and *N*-benzylsulfamide (13 mg, 0.068 mmol). After the reaction mixture was allowed to stir for 22 h at rt, EtOAc (20 mL) and aqueous saturated NH_4_Cl (20 mL) were added. After separation, the aqueous phase was extracted with EtOAc (20 mL). The combined organic phases were dried (Na_2_SO_4_) and concentrated. After gradient column chromatography (25% → 50% EtOAc in heptane), product **E** was obtained as an inseparable mixture of the title compound and *N*-benzylsulfamide (3.2/1 mass ratio) (14.4 mg). ^1^H NMR (400 MHz, CDCl_3_) δ (ppm) 7.38–7.27 (m, 5H), 5.99 (bs, 1H), 5.01 (t, *J* = 6.2 Hz, 1H), 4.20 (s, 2H), 4.85–4.70 (m, 2H), 4.13 (d, *J* = 8.12 Hz, 2H), 3.15 (q, *J* = 6.50, 2H), 2.35–2.15 (m, 6H), 2.09 (t, *J* = 7.4 Hz, 2H), 1.65–0.75 (m, 13H).

Compound **F.** To a solution of BCN-OSu carbonate (430 mg, 1.48 mmol) in DCM (20 mL) was added a solution of 5-aminopentan-1-ol (152 mg, 1.47 mmol) in DCM (4 mL) and Et_3_N (619 μL, 449 mg, 4.44 mmol). The mixture was stirred for 1.5 h at rt after which a saturated aqueous solution of NaHCO_3_ was added (40 mL). After separation, the organic layer was dried (Na_2_SO_4_) and concentrated. The residue was purified by gradient column chromatography (EtOAc/heptane 1/1 → 3/1). The product alcohol was obtained as a colorless sticky liquid (356 mg, 1.27 mmol, 81%). ^1^H NMR (400 MHz, CDCl_3_) δ (ppm) 4.68 (s, 1H), 4.14 (d, *J* = 8.0 Hz, 2H), 3.65 (dd, *J* = 11.7, 6.3 Hz, 2H), 3.19 (dd, *J* = 13.2, 6.7 Hz, 2H), 2.35–2.15 (m, 6H), 1.66 – 1.30 (m, 7H), 1.02–0.88 (m, 2H).

To a solution of the alcohol obtained above (51 mg, 0.18 mmol) in DCM (10 mL) was added chlorosulfonyl isocyanate (16 μl, 25 mg, 0.18 mmol). After the mixture was stirred for 40 min, Et_3_N (75 μl, 55 mg, 0.54 mmol) and benzylamine (19 μl, 19 mg, 0.18 mmol) were added. The mixture was stirred for an additional 1.5 h and quenched through addition of an aqueous solution of NH_4_Cl (sat). After separation, the aqueous layer was extracted with DCM (20 mL). The combined organic layers were dried (Na_2_SO_4_) and concentrated. The residue was purified by gradient column chromatography (20% → 50% EtOAc in pentane) and product **F** was obtained as colorless thick oil (57 mg, 0.12 mmol, 67%).^1^H NMR (400 MHz, CDCl_3_) δ (ppm) 7.41–7.28 (m, 5H), 5.55 (s, 1H), 4.75 (s, 1H), 4.29­–4.24 (m, 2H), 4.20–4.08 (m, 2H), 3.19 (dd, *J* = 13.4, 6.6 Hz, 2H), 2.37–2.16 (m, 6H), 1.74–1.31 (m, 9H), 0.94 (t, *J* = 9.7 Hz, 2H).

Compound **G.** Under an inert atmosphere, compound **F** (93 mg, 0.19 mmol) was dissolved in anhydrous THF (10 mL). PPh_3_ (49 mg, 0.19 mmol) and MeOH (50 µL, 1.23 mmol) were added and the mixture was cooled to 0 °C. A solution of DIAD (37 µL, 0.19 mmol) in anhydrous THF (5 mL) was slowly added and the mixture was allowed to reach rt, after which the reaction was stirred for 18 h and subsequently concentrated. Gradient column chromatography (20 → 50% EtOAc in heptane) yielded product **G** as colorless thick oil. ^1^H NMR (400 MHz, CDCl_3_) δ (ppm) 7.39–7.26 (m, 5H), 5.94–5.84 (m, 1H), 4.80–4.64 (m, 1H), 4.19 (d, *J* = 6.4 Hz, 2H,), 4.13 (d, *J* = 7.4 Hz, 2H), 4.08 (t, *J* = 6.5 Hz, 2H), 3.17 (q, 2H, *J* = 6.5 Hz), 3.12 (s, 3H), 2.35–2.14 (m, 6H), 1.80–1.65 (m, 13H).

Compound **H.** To a solution of the BCN-alcohol (1.5 g, 10 mmol) in DCM (150 mL), under a N_2_ atmosphere, was added CSI (0.87 mL, 1.4 g, 10 mmol), Et_3_N (2.8 mL, 2.0 g, 20 mmol) and 2-(2-aminoethoxy)ethanol (1.2 mL, 1.26 g, 12 mmol). The mixture was stirred for 10 min and quenched through addition of aqueous NH_4_Cl (sat., 150 mL). After separation, the aqueous layers was extracted with DCM (150 mL). The combined organic layers were dried (Na_2_SO_4_) and concentrated. The residue was purified with column chromatography. Product alcohol was obtained as slightly yellow thick oil (2.06 g, 5.72 mmol, 57%). ^1^H NMR (400 MHz, CDCl_3_) δ (ppm) 6.0 (bs, 1H), 4.28 (d, *J* = 8.2 Hz, 2H), 3.78–3.73 (m, 2H), 3.66–3.61 (m, 2H), 3.61–3.55 (m, 2H), 3.34 (t, J = 4.9 Hz, 2H), 2.37­–2.15 (m, 6H), 1.64–1.48 (m, 2H), 1.40 (quintet, *J* = 8.7 Hz, 1H), 1.05–0.92 (m, 2H).

To a solution of the intermediate alcohol (130 mg, 0.36 mmol) were subsequently added CSI (31 µL, 51 mg, 0.36 mmol), Et_3_N (151 µL) and 2-(2-aminoethoxy)ethanol (36 µL, 38 mg, 0.36 mmol). After 15 min, water (20 mL) was added and after separation, the aqueous layer was acidified with 1 M aq. HCl to pH 3 and extracted with DCM (20 mL). The DCM layer was dried and concentrated. After column chromatography, the product bis-sulfamide alcohol was obtained as colorless oil (87 mg, 0.15 mmol, 42%). ^1^H NMR (400 MHz, CDCl_3_) δ (ppm) 6.15–5.95 (m, 2H), 4.40–4.32 (m, 2H), 4.31 (d, *J* = 8.3 Hz, 2H), 3.85–3.55 (m, 10H), 3.45–3.25 (m, 4H), 2.40–2.15 (m, 6H), 1.65–1.47 (m, 2H), 1.40 (quintet, *J* = 8.7 Hz, 1H), 1.06–1.92 (m, 2H).

To a solution of the intermediate bissulfamide alcohol (63 mg, 0.11 mmol) in DCM (10 mL) were subsequently added *p*-nitrophenyl chloroformate (22 mg, 0.11 mmol) and Et_3_N (46 µL, 33 mg, 0.33 mmol). After 20 h, benzylamine (22 µL, 21.6 mg, 0.20 mmol) was added to the reaction mixture. The mixture was stirred for an additional 24 h where after the mixture was concentrated and the residue was purified by gradient column chromatography (1st col. 0 → 20% MeOH in DCM, 2nd col. 0 → 8% MeOH in DCM). Product **H** was obtained as a colorless film (18 mg, 0.026 mmol, 23%). ^1^H NMR (400 MHz, CD_3_OD) δ (ppm) 7.38–7.18 (m, 5H), 4.31–4.22 (m, 6H), 4.22–4.16 (m, 2H), 3.70–3.63 (m, 4H), 3.63–3.54 (m, 4H), 3.34 (s, 1H), 3.24­–3.15 (m, 4H), 2.30–2.10 (m, 6H), 1.68–1.52 (m, 2H), 1.42 (quintet, *J* = 8.7 Hz, 1H), 1.02–0.90 (m, 2H).

**Intact protein analysis - Sample preparation**

Protein reduction was performed for 30 minutes in 10 mM DTT at 56 °C. Samples were diluted 1:1 in 2% formic acid prior to analysis.

**Liquid chromatography – mass spectrometry**

Protein separations were performed using a UHPLC nanoflow liquid chromatography (Bruker Daltonics nano advance) coupled online to an ultra high resolution quadrupole time-of-flight mass spectrometer (Bruker Daltonics maXis 4G ETD) via an axial desolvation vacuum assisted electrospray source (Bruker Daltonics captive sprayer). Proteins were loaded on the trap column (Dionex PepSwift, 0.2 × 5 mm) in 3 minutes at a flowrate of 5000 nL/min using 0.1% formic acid. Proteins were separated on a 0.2 × 150 mm monolithic particle column (Michrom 8μm 4000Å PLRP-S) at 50°C using a linear gradient of 20 to 50% acetonitrile and 0.1% formic acid at a flowrate of 1000 nl/min. Desolvation and ionization of peptides eluting from the column was performed using 6 L/min nitrogen gas at 180 °C and 1600 V capillary voltage. The mass spectrometer was calibrated externally using Agilent tuning mix (G1969-85000) and used lockmass calibration at 1221.9906 m/z (Agilent G1982-85001). The mass spectrometer was programmed to acquired spectra in the range of 500-4200 m/z at 1 Hz with the following settings: 400 Vpp Funnel RF, 10 eV isCID, 400 Vpp Multipole RF, 8 eV Quadrupole ion energy, 510 m/z Low mass, 10 eV Collision cell energy, 3500 Vpp Collision RF, 110 μs Transfer time, 450 Vpp Ion cooler RF and 18 μs Pre pulse storage.

**Data processing**

All data were processed in Data Analysis software. After lock mass calibration, both the light- and heavy-chain MS spectra were averaged over each chromatographic peak, respectively. Averaged spectra were deconvoluted using the maximum entropy algorithm in combination with the SNAP peak picking algorithm for the light chain spectrum or Sum peak picking for the heavy chain spectrum.

**Proteolytic peptide analysis – *Sample preparation***

From each sample, five μg of protein was subjected to in-solution tryptic digestion. Briefly, reduction was performed in 10 mM DTT for 30 minutes at 56 °C. Alkylation of reduced cysteines (carbamidomethylation) was performed using 50 mM chloroacetamide. Protein digestion was first performed by addition of 0.5 µg LysC peptidase and incubation for 3 hours at 37 °C. Next, 800 ng trypsin was added to the sample and incubated O/N at 37 °C. Resulting proteolytical peptides were concentrated and desalted using stop-and-go elution tips.

**Liquid Chromatography *– Tandem mass spectrometry***

Peptide separations were performed using a UHPLC nanoflow liquid chromatograph (Bruker Daltonics nano advance) coupled online to a high capacity ion trap (Bruker Daltonics amaZon speed ETD) via an axial desolvation vacuum assisted electrospray source (Bruker Daltonics captive sprayer). Peptides were loaded onto the trap column (Dionex PepSwift, 0.2 × 5 mm) in 3 minutes at a flowrate of 5000 nL/min using 0.1% formic acid. Peptides were separated on a 0.1 × 250 mm monolithic column (Dionex PepSwift) at 60°C using a linear gradient of 5 to 25% acetonitrile and 0.1% formic acid at a flowrate of 800 nL/min. Desolvation and ionization of peptides eluting from the column was performed using 3 L/min nitrogen gas at 150 °C and 1300 V capillary voltage. The mass spectrometer was programmed to acquire a single survey spectrum (MS) with subsequent data dependent fragmentation analysis (MS/MS) of the top 6 most abundant ions. Survey spectra were acquired at enhanced resolution mode and used the following instrument settings: 50 ms maximum accumulation time, 500.000 ICC target, tune at 1000 m/z, 5 spectra averages. Fragmentation spectra were acquired in extreme scan mode with autoselect fragmentation mode enabled which switches between collision induced dissociation (CID) and electron transfer dissociation (ETD) fragmentation methods based on the mass-to-charge ratio and charge state of the precursor ion. The following instrument settings were used for fragmentation scans: 500.000 ICC target, 200 ms maximum accumulation time, SPS enabled, 70% CID energy and 0.2 min dynamic exclusion.

**Preparation and characterization of trastuzumab-1**

Remodeling of trastuzumab of DAR2 ADCs **3a-d** and **4a-b** was performed according to the reported procedure based on GalNAz and GalT(Y289L)^[[1]](#endnote-1)^. For preparation of trastuzumab DAR4 ADC **6**, a similar procedure was applied but with 6-azidoGalNAc substrate instead of GalNAz. In short, trimmed trastuzumab (10 mg/mL), obtained by treatment with an EndoS mutant, was incubated with UDP-6-azidoGalNAc (1 mM, commercially available from GlycoHub) in 10 mM MnCl_2_ and 25 mM Tris-HCl pH 8.0 and the reaction was incubated overnight at 30 °C. Trastuzumab-**1** was purified from the reaction mixture on a HiTrap MabSelect SuRe 5 ml column (GE Healthcare) using an AKTA purifier-10 (GE Healthcare). The eluted IgG was immediately neutralized with 1.5 M Tris-HCl pH 8.8 and dialyzed against PBS pH 7.4. Next, the IgG was concentrated using an Amicon Ultra-0.5, Ultracel-10 Membrane (Millipore) to a concentration of 23 mg/mL. Mass spectral analysis of a IdeS-digested sample showed one main of the Fc/2-fragment (~90%) belonging to one major product (observed mass 24363 Da, Fc/2 fragment), corresponding to core 6-N_3_-GalNAc-GlcNAc(Fuc)-substituted trastuzumab.

**Preparation and characterization of brentuximab-1**

Glycan trimming of cAC10/brentuximab (14.5 mg/mL), obtained via transient expression in CHO K1 cells performed by Evitria (Zurich, Switzerland), was incubated with EndoS mutant (1 w/w%) in 25 mM Tris pH 7.5 with 150 mM NaCl for approximately 16 hours at 37 °C. The trimmed IgG was dialyzed against 3x1 L of 25 mM Tris-HCl pH 8.0. Mass spectral analysis of a fabricator-digested sample showed three peaks of the Fc/2-fragment belonging to one major product (observed mass 24105 Da, approximately 80% of total Fc/2 fragment), corresponding to core GlcNAc(Fuc)-substituted brentuximab, and two minor products (observed masses of 23959 and 24233 Da, approximately 5 and 15% of total Fc/2 fragment), corresponding to core GlcNAc-substituted brentuximab and core GlcNAc(Fuc)-substituted brentuximab with C-terminal lysine.

Trimmed brentuximab (10 mg/mL), was incubated with the substrate 6-N_3_-GalNAc-UDP (2.5 mM, commercially available from GlycoHub) and 0.5 mg/mL TnGalNAcT (5 w/w%) as described for trimmed trastuzumab above. Mass spectral analysis of a IdeS-digested sample showed three peaks of the Fc/2-fragment belonging to one major product (observed mass 24333 Da, approximately 80% of total Fc/2 fragment), corresponding to core 6-N_3_-GalNAc-GlcNAc(Fuc)-substituted brentuximab, and two minor products (observed masses of 24187 and 24461 Da, approximately 5 and 15% of total Fc/2 fragment), corresponding to core 6-N_3_-GalNAc-GlcNAc-substituted brentuximab and core 6-N_3_-GalNAc-GlcNAc(Fuc)-substituted brentuximab with C-terminal lysine.

**Aggregation studies**

HydraSpace™ *vs* PEG-only DAR2 ADC

Aggregation behaviour of ADCs **3c** and **3d** was determined by incubation at a concentration of 1 mg/mL in Na-citrate pH 5.0, 150 mM NaCl at 37 °C. After 0, 2, 7, 10 and 14 days samples were analyzed using a superdex200 pc 3.2/30 (GE Healthcare) on an AKTA Purifier-10 (GE Healthcare). The percentage of aggregate peaks compared to the total peak area was quantified using the absorbance at 215 nm.

DAR4 ADC trastuzumab-**6** *vs* Kadcyla

Human serum (Sigma Aldrich) was incubated with protein A at 4 °C for 2 hours and subsequently removed to obtain IgG-depleted human serum. Trastuzumab-based ADCs and trastuzumab were incubated with human serum at 0.1 mg/mL and 37 °C. Prior to analysis on size-exclusion chromatography, individual samples were prepared by a small-scale protein A incubation step in order to remove human serum additives and/or proteins. The individual trastuzumab-based ADCs were eluted from protein A beads by a 5 minute incubation step with glycine pH 2.7 , neutralized and injected on the SEC-column (Waters, X-bridge).

**Supplementary Table 1. Mass spectrometric characterization of ADCs obtained by conjugation of trastuzumab-1 and brentuximab-1:**

| **Antibody** | **Azidosugar**  **substrate** | **BCN-spacer-payload** | **MS main peak (Da)** |
| --- | --- | --- | --- |
| Trastuzumab-**1** | GalNAz | **3a** | 50982 (heavy chain) |
|  | GalNAz | **3b** | 50938 (heavy chain) |
|  | GalNAz | HS-vcPABC-beta-Ala-May | 51260 (heavy chain) |
|  | GalNAz | PEG-vcPABC-beta-Ala-May | 51186 (heavy chain) |
|  | GalNAz | **4a** | 51289 (heavy chain) |
|  | GalNAz | **4b** | 51237 (heavy chain) |
|  | 6-N_3_-GalNAc | **6** | 27331 (Fc/2-fragment) |
| Brentuximab-**1** | 6-N_3_-GalNAc | **5a** | 25844 (Fc/2-fragment) |
|  | 6-N_3_-GalNAc | **5b** | 25853 (Fc/2-fragment) |
|  | 6-N_3_-GalNAc | **7** | 27124 (Fc/2-fragment) |

**NMR Spectra**

Alcohol precursor for **3a**

PNP carbonate precursor for **3a**

Alcohol precursor for compound **3c**

Spectrum after PNP activation of **3c**

Precursor for compound **3d**

Precursor for Compound **5b** (alcohol)

Precursor for Compound **5b** (carbonate)

Intermediate diol carbonate for Compound **6**

Diol precursor for compound **7**

Carbonate precursor for compound **7**

BCN-aminoheptanoic acid NHS ester

Compound **D**

*tert*-butyl *N*-benzylsulfamoylcarbamate

*N*-benzylsulfamide

BCN-aminoheptanoic acid

Compound E/ *N*-benzylsulfamide mixture

Alcohol precursor for compound **F**

mixture

Compound **F**

mixture

Compound **G**

mixture

Bissulfamide alcohol

mixture

Compound **H**

1. Van Geel R., Wijdeven M.A., Heesbeen R., Verkade J.M., Wasiel A.A., van Berkel S.S., van Delft F.L.. Chemoenzymatic conjugation of toxic payloads to the globally conserved N-glycan of native mAbs provides homogeneous and highly efficacious antibody−drug conjugates. *Bioconj Chem.* **2015**, *26*. 2233-2242. [↑](#endnote-ref-1)
